# Morphological and histochemical characteristics of the foregut, midgut, and hindgut, and their alterations during ovarian development in female freshwater prawn, *Macrobrachium rosenbergii*

**DOI:** 10.1007/s00441-024-03948-w

**Published:** 2025-01-13

**Authors:** Warinthip Vetkama, Ruchanok Tinikul, Prasert Sobhon, Yotsawan Tinikul

**Affiliations:** 1 Department of Anatomy, Faculty of Science, Mahidol University, 272 Rama VI Road, Ratchathewi District, Bangkok, 10400 Thailand; 2 Department of Biochemistry and Center for Excellence in Protein and Enzyme Technology, Faculty of Science, Mahidol University, Bangkok, 10400 Thailand

**Keywords:** *Macrobrachium rosenbergii*, Digestive organs, Histochemical structures, Ovarian development, Giant freshwater prawn

## Abstract

The anatomical, histological, and histochemical characteristics of the foregut (FG), midgut (MG), and hindgut (HG), as well as their alterations during the ovarian cycle in female prawns, *Macrobrachium rosenbergii*, were investigated. The esophagus (ESO), cardia (CD), and pylorus (PY) are the main components of the FG. An epithelium (Ep) with thick cuticle (Cu) layers lining the ESO, and the ESO is encircled by the ESO glands. The CD has a thick musculature, whereas the Ep of the PY are characterized by numerous villi and columnar Ep cells with a thinner layer of Cu. The inner longitudinal (LM) and the outer circular (CM) muscles were both present in the PY. The MG is lined by Ep cells which are connected to the basement membrane, and it lacks Cu. Microvilli, and subapical vacuoles are visible on the apical surface of Ep cells of the MG. The outermost layer is characterized by a dense strip of elastic fibers and a cluster of collagen fibers. The HG has the Ep cells with a thin Cu layer, and the HG glands form a rosette-like structure. The HG is surrounded by the CM and the LM fibers. The reactivities of Periodic Acid Schiff and Alcian Blue in these digestive organs altered throughout the ovarian cycle, and this was supported by the increased expression of mucin levels as ovarian maturation progressed. Our results offer novel and significant insights into the anatomical and histochemical structures of these digestive organs, and demonstrate a significant correlation between ovarian development and feeding in the female prawn, *M. rosenbergii*.

## Introduction

The giant freshwater prawn, *Macrobrachium rosenbergii*, is one of the most commercially valuable crustacean species in Asian countries, including China, Indonesia, Philippines, Vietnam, and Thailand. They are in high demand for both local consumption and international markets, which contribute greatly to the income of the prawn farmers as well as substantial economic and societal progress in the countries (Lin et al. 1988; New 2008; New and Nair 2012; Tinikul et al. 2023). However, diseases, environmental impact, high stocking density, low food consumption, and nutrient deficiencies impose some limitations on the farming and production of this prawn (Dierberg and Kiattisimkul 1996; Racotta et al. 2003), which result in poor growth rates of post-larvae and juvenile prawns, as well as reduced reproductive performance of female broodstocks (Dash et al. 2016; Sagi and Aflalo 2005; Rahman et al. 2022). Enhancing feeding performance is one of the important strategies required for female broodstocks to gain more nutrients and energy to promote ovarian maturation and vitellogenesis (Barros and Valenti 2003; Hossain and Paul 2007; Griffen 2018). Hence, a basic understanding of the anatomy and physiology of the digestive system is essential for comprehending the nutrition uptake and processing of this prawn species, and other decapod species (Ceccaldi 1989; Vogt 1996). This will help to further develop aquaculture for this prawn species (Zambonino-Infante et al. 2008).

In decapod crustaceans, the digestive system plays important functions in digestion, food processing, absorption, and the storage of nutrients (Ceccaldi 1989; Štrus et al. 2019; Vogt 1996, 2021). The digestive organs consist of three main parts: the cuticle-lined foregut (FG), the midgut (MG), and the hindgut (HG), which lacks a cuticle (Icely and Nott 1992; Vogt 2021). The mouth (Mo) opens directly into the foregut (FG), including the esophagus (ESO), a short tubular structure, and the esophageal gland (ESOG), which secretes mucins to lubricate and facilitate the passage of food particles and materials into the MG (Icely and Nott 1992; McGaw et al. 2013). Mucins (MUCs) are the main components of the mucus produced by digestive cells, and MUCs have important roles in nutrient absorption and immune responses in aquatic animals (Duan et al. 2018; Wang et al. 2022). In fish and crustaceans, MUC2 and MUC19 in the intestine have roles in nutritional digestion and absorption, mucus production, maintaining the intestinal barrier, and promote the innate immune response (Wang et al. 2012; Duan et al. 2018). The digestive process proceeds in the mixture of mucin and food stored within the ESO, which facilitates the chemical decomposition of food that is subsequently filtered in the FG chamber before passing into the MG, which is responsible for absorbing nutrients, and finally into the HG, which is responsible for excreting the remaining residues as feces (Vogt 1996, 2021).

In female decapod crustaceans, such as *M. rosenbergii*, vitellogenesis is an essential physiological step for ovarian development (Jayasankar et al. 2020; Tinikul et al. 2023). The ovarian cycle of female *M. rosenbergii* is categorized into four stages according to ovary size, color, and histology: stage I (spent stage), stage II (proliferative stage), stage III (premature stage), and stage IV (mature stage). The ovarian cycle endures around 40 days. The morphological and histological structures of these ovarian phases were identified based on the previously established standards (Tinikul et al. 2023; Vetkama et al. 2024). Ovarian maturation of female decapod crustaceans is an energy-intensive process (Griffen 2018; Tinikul et al. 2022); consequently, increased food intake is required to acquire more nutrients and energy necessary for vitellogenesis and ovarian development (Adiyodi 1988; Griffen 2018). The stored nutrients in the hepatopancreas are mainly utilized for metabolic processes in the ovary, including the maturation of oocytes and the synthesis of vitellogenin (Damrongphol et al. 1991; Cavalli et al. 2000; Vogt 2021). The nutritional intake and digestive organ performance of female crustaceans directly influence the development and composition of the ovary. Thus, there appears to be a dynamic correlation between nutritional composition, changes in digestive organs, and ovarian development (Kung et al. 2004; Wu et al. 2014; Fang et al. 2023). Additionally, the changes in the morphological structure and histochemical characteristics of the digestive organs are also correlated with the lifecycle and feeding status (Icely and Nott 1992; Kawamura 2018; Rocha 2018), as shown in several decapod crustaceans, including *Xuntho bidentatus* (Erric Babu et al. 1979b), *Scylla serrata* (Monin et al. 1974), *Procambarus clarkii* (Miyawaki et al. 1961), *Malacostraca brachydactyla* (Castejón et al. 2021), and *Litopenaeus vannamei* (Muhammad et al. 2012).

The intestinal characteristics, particularly villi length, villi height, diameter of gland are essential components of the digestive tract that indicate the digestive function (Yamauchi 2007; Moniello et al. 2019). In decapod crustaceans, morphological alterations observed in the midgut region, specifically the increase of intestinal villi height and number of cells, improved the intestinal epithelial barrier function, and surface area for nutritional absorption (Goh et al. 2023). A higher surface area and height of the intestinal villus facilitate enhanced nutrition absorption in the digestive tract (Moniello et al. 2019). High secretory activity was associated to ovarian development and vitellogenesis in the crab, *Paratelphusa* sp. (Sarika et al. 2014). Taken together, there may be a correlation between digestive structures and reproductive performance in female crustacean species. However, the detailed morphological, histological, and histochemical characteristics of the FG, MG, and HG of the female prawn, *M. rosenbergii* during the ovarian cycle have not yet been investigated. Thus, we question whether there are morphological and histochemical changes in digestive organs that may be related to the ovarian maturation cycle in this female *M. rosenbergii*. These changes may reflect the functional connection between feeding and nutritional processing by the digestive tract which may serve ovarian maturation in this crustacean species.

In the present study, we are the first to examine the detailed anatomical, histological, and histochemical characteristics and the changes of the FG, MG, and HG, during the ovarian cycle of female prawns, *M. rosenbergii*. Additionally, we investigated the changes in MUCs expression by immunohistochemistry and qPCR, and studied changes in glycoproteins with histochemical techniques. These changes would be expected to increase lubrication and enhance nutrient absorption, which, in turn, would enhance ovarian development. Our study provides the first important evidence for understanding the basic knowledge of the morphological, histological, and histochemical structures of the FG, MG, and HG, as well as verifying the possible correlation between feeding and ovarian development in female prawns, *M. rosenbergii*. Importantly, our findings could have implications for aquaculture of this economically important prawn species and may lead to improved methods for a more successful production of this prawn.

## Materials and methods

### Experimental animals and acclimatization

Female *M. rosenbergii* prawns, with an average weight of 34–40 g each, were acquired from local markets, Ratchaburi Provinces, in Thailand. The prawns were cultured in circular fiberglass tanks measuring approximately 1.50 m in diameter, with a filtered water level of around 0.80 m and aeration was provided during the entire day. The water temperature was maintained at about 27–28 °C. One-third of the water was replaced every two days. The prawns were given commercial food pellets twice daily (Charoen Pokphand Group, Thailand). At least 7–8 plastic cages were placed in each tank for molting animals to seek shelter, and the prawns were acclimated to a 12:12 h light–dark cycle.

### Anatomical nomenclature of the foregut (FG), midgut (MG), and hindgut (HG)

We adopted similar conventional names for the FG, MG, and HG as other *Macrobrachium* species (Ruiz et al. 2020), and other malacostracan crustacean species (Ceccaldi 2006; Štrus et al. 2019; Vogt 2019) with some modifications.

### Processing tissues for internal morphology and light microscopy

All prawns were euthanized on ice for a minimum of 10–15 min until no movement was detected. The female prawns were bisected along an imaginary line connecting the left and right mandibles, as well as at the cephalothorax-abdominal junction. The anterior portion was dissected to separate the FG, including the esophagus (ESO), esophageal gland (ESOG), cardia (CD), and pylorus (PY). Additionally, the posterior portion was dissected to locate the MG and HG. The digestive organs were washed in phosphate-buffered saline (PBS), and then placed in a fixative solution with 4% paraformaldehyde in 0.1 M PBS, pH 7.4, for 12 −14 h. They were then rinsed three times with 0.1 M PBS, and the tissues were dehydrated using a series of ethanol solutions, cleaned with xylene twice, and then placed in liquid paraffin before being embedded in paraffin blocks. The tissue slices were cut at a thickness of 5–6 µm and placed on slides treated with a 3-aminopropyl triethoxy-silane solution (Sigma Co., St. Louis, MO, USA). The sections were deparaffinized in three xylene changes for 5 min each, followed by rehydration in a series of graded ethanol with two final changes of distilled water for 15 min. Subsequently, they were stained with hematoxylin and eosin (H & E). The morphology and histological structures of digestive organs were carefully observed using a Nikon E600 light microscope. Images were acquired with a Nikon DXM digital camera and ACT-1 software.

### Semithin sectioning

The FG, MG and HG were obtained from each of the four ovarian stages (at least n = 3–4 animals). The methods used were based on those previously described (Nontunha et al. 2023) with some modifications. The tissues were dissected and promptly preserved using a solution containing 4% paraformaldehyde and 2.5% glutaraldehyde. The tissues were rinsed three times with 0.1 PBS at pH 7.4, for 5 min each time. The tissues were post-fixed with 1% osmium tetroxide after washing to preserve phospholipids in the cell membrane for 1 h at 4 ºC in a dark chamber. The sections were dehydrated using a sequence of cold ethanol (50%, 70%, 80%, 90%, 95%, and 6 × 100%) for 15 min each in a dark chamber with gentle shaking. Following dehydration, each tissue was immersed in propylene oxide as an intermediate solution, then the solvent was then replaced with 1 to 1 by volume of propylene oxide: araldite mixture. The tissues were sequentially immersed in propylene oxide and araldite at different ratios for certain durations and followed by overnight incubation with gentle shaking. Tissues were infiltrated with araldite and incubated for 6 h in a fumed hood to remove propylene oxide. Subsequently, the tissues were embedded in araldite resins (Araldite 502, DDSA, DMP-30, electron microscopy science), and placed in a mold. They were then incubated at 45°C and 60°C in an oven for 2 days to polymerize the araldite resin. The tissue-containing resin blocks were sectioned using an ultramicrotome, Leica Ultracut UCT25. The semithin sections were stained with 1% methylene blue and observed under a light microscope (Nikon DXM1200F) equipped with a CCD camera.

### Histochemical characteristics of the FG, MG, and HG by Periodic Acid Schiff (PAS) and Alcian Blue (AB)

The FG, MG, and HG during ovarian stages I-IV (at least n = 10 animals per stage) were collected and fixed with 4% paraformaldehyde in 0.1 M PBS, pH 7.4, for 12 h at 4 ºC. The paraffin-embedded of tissue were cut at a thickness of 5 μm, and the sections were further immersed at xylene three times, for 5 min each, and then immersed with a graded series of ethanol (100%, 95%, 90%, 80%, 70%), for 3 min each. Sections were stained to Periodic Acid-Schiff reaction (PAS) (Abcam) for neutral glycoproteins and/or glycogen staining, and Alcian blue (AB) (Abcam) for acid glycoproteins staining (carboxylated and sulfated), based on the previous descriptions (Layton and Bancroft 2019; Ruiz et al. 2020). For staining with AB and PAS, sections were immersed with distilled water for 15 min, and then incubated with AB at pH 2.5 solution for 30 min in a moist chamber. After incubating, the sections were incubated with sodium tetraborate hydroalcoholic solution, for 10 min, in a moist chamber. After that, the sections were washed with distilled water for 1 min, and then incubated with periodic acid solution for 10 min. The sections were washed with distilled water for 3 dips, and then incubated with Schiff Reagent Hotchkiss McManus, for 45 min, in a moist chamber at dark room. The sections were washed with distilled water by 5 dipping, and incubated with potassium metabisulphite solution for 2 min. The sections were drained without washing, and incubated with fixation solution for 2 min. After that, the sections were rinsed in distilled water, and counterstained nuclei with Mayer’s hematoxylin. Subsequently, the sections were washed in running tap water for 5 min, and dehydrated through a graded series of ethanol (70%, 80%, 90%, 95%, and 100%) for 3 min each, and cleared in xylene three time (5 min each). Finally, sections were mounted by Permount (Bio-Optica, Milan, Italy).

### Histochemical analyses of the FG, MG, and HG by Verhoeff–Van Gieson staining

Tissue sections of the FG, MG, and HG were deparaffinized and hydrated in distilled water. The slides were immersed in a working elastic staining solution for 15 min, and then washed with running tap water until no excess stain was visible on the slide. The slides were immersed in a differentiating solution many times and then washed with tap water. They were examined under a microscope to ensure proper differentiation. The slides were washed in tap water and then immersed in a sodium thiosulfate solution for 1 min. The sections were washed with tap water, then stained with Van Gieson's Solution, which was used to stain connective tissue (Abcam) for 2–5 min, and finally rinsed in two changes of 95% ethanol. The slides were dehydrated in absolute alcohol, and mounted in mounting media.

### Measurements of defined morphological characteristics of digestive organs

The epithelial cell (Ep) diameter, villi height, villi width, crypt depth, number of cells, and gland diameter were measured utilizing Image J software (NIH, RRID: SCR_003073, Bethesda, MD, USA; available on the internet: http://rsb.info.nih.gov/ij/). The methods used were based on the previous description (Guo et al. 2016; Liu et al. 2023) with some modifications. We quantified the Ep cells and villi in a minimum of ten individual female prawns at stages I-IV. The Ep cells (at least 30 cells) in the ESO, CD, and PY areas, as well as the Ep cells lining the villi (at least 20–30 cells per Vi) were quantified. In the MG, the Ep cells and villi (at least 10 animals per stage) were measured., while villi and gland cells at the stages I-IV (at least 10 animals per stage) in the HG were measured. At least ten villi of HG and Ep cells were measured from a minimum of 20 acini of HG glands. These experiments were performed in triplicate.

### Expression of *MUC* transcript in the FG, MG, and HG using reverse transcription real-time quantitative PCR (RT-qPCR)

The FG, MG, and HG at different ovarian stages, were collected (n = at least 15 females/digestive part/ovarian stage). They were promptly frozen in liquid nitrogen and stored at −80 °C. RT-qPCR was conducted following a previously established procedure (Vetkama et al. 2024), with some modifications. RNA was isolated using TRIzol™ reagent (Invitrogen, Carlsbad, CA, USA) following the manufacturer's guidelines. The samples were subjected to chloroform treatment and centrifuged at 12,000 rpm for 10 min at 4 °C. RNA was precipitated using isopropanol by incubating the samples at −80 °C for 15 min followed by centrifugation at 12,000 rpm for 10 min at 4 °C. The pellet was rinsed twice with 70% ethanol (v/v). The desiccated RNA pellet was dissolved in DEPC-treated water and stored at −80 °C. RNA concentration and quality were assessed by 1% agarose gel electrophoresis and spectrophotometry using a NanoDrop 1000 (Thermo Fisher Scientific). The A260/A280 and A260/A230 ratios of all RNA samples were suitable for RT-qPCR. The DNase treatment used 800 ng of RNA and 1 unit of DNaseI, conducted in 1 DNase reaction buffer (Promega, Wisconsin, USA). After incubating for 30 min at 37 °C, the process was halted by adding 1 L of DNase stop solution. The samples were incubated at 65 °C for 10 min to deactivate DNase. Approximately 320 nanograms of RNA was converted into first-strand cDNA using the ImProm-II III Reverse Transcriptase system (Promega, Wisconsin, USA), with the use of random hexamer primers. Subsequently, qPCR was conducted in a 20 μl reaction system including 2 μl of undiluted cDNA from reverse transcription, 10 μl of KAPA SYBR FAST qPCR Master Mix (2) from KAPA Biosystems, USA, and 200 nM of forward and reverse primers. The experiments were conducted using the CFX96 TouchTM Real-Time PCR Detection system from Bio-Rad Laboratories, USA, following specific thermal cycling conditions: 95 °C for 3 min, 40 cycles of 95 °C for 3 s, 60 °C for 20 s, and 65 °C for 5 s. Melting curve analysis was performed at 95°C for 5 min, followed by 55°C to 95°C, with continuous fluorescence reading at every 0.5°C increment to confirm the specificity of the PCR product. Every qPCR experiment contained a negative control lacking cDNA. Experiments were conducted three times. The relative expression level of *MUC19* transcript was calculated using the 2^−ΔΔCt^ method (Livak and Schmittgen 2001) to evaluate the fold-changes in *MUC19* transcript abundance in these digestive organs compared to stage I values. The relative expression of *MUC19* mRNA normalized to that of the *elongation factor 1 alpha* (*EF-1α*) gene. The qPCR primers used in this work are detailed in Table 1.

**Table 1:** Sequences of primers used for RT-qPCR in female *M. rosenbergii*

**Primer**	**Direction**	**Nucleotide sequence (**5´- 3´)
MrMUC-F	Forward	5´ CAAGATACTCAGTGCCAAGT 3´
MrMUC-R	Reverse	5´ GAAGGAGTGTCACCAAAGAT 3´
EF-1α-F	Forward	5´GGTGCTGGACAAGCTGAAGGC 3´
EF-1α-R	Reverse	5´CGTTCCGGTGATCATGTTCTTGATG 3´

### Immunolocalization of mucins (MUCs) in the FG, MG, and HG

The existence and expression of MUCs-like-ir in the FG, MG, and HG were examined during the ovarian cycle (at least n = 10 prawns per stage). The tissues were fixed by immersing in a solution of 4% paraformaldehyde in 0.1 M PBS at 4 °C for 12 h. After fixation and paraffin embedding, the tissues were cut at a thickness of 5–6 μm, and then placed on slides treated with a 3-aminopropyl triethoxy-silane solution (Sigma-Aldrich Co). Next, the sections were immersed in a 1% glycine solution in PBS for 15 min, then transferred to PBS for 5 min. To prevent nonspecific binding, the sections were incubated in a blocking solution consisting of 10% normal goat serum (NGS) and 0.4% triton-X in PBS (PBST) for 2 h in a humidified chamber. The sections were incubated with the primary antisera, goat anti-MUC19, diluted 1:200 (Abcam), rabbit anti- MUC2, diluted 1:400 (Abcam), or mouse anti-E-Cadherin antibodies (diluted 1:100, Santa Cruz Biotechnology). The tissue sections were incubated in the primary antisera in a blocking solution at room temperature overnight. Afterward, the samples were rinsed three times with PBST and then exposed to the secondary antibodies: Alexa 488-conjugated goat anti-rabbit IgG or Alexa 568-conjugated goat anti-mouse IgG or Alexa 647-conjugated donkey anti-goat IgG (Molecular Probes, Eugene, USA) at a dilution of 1:500 in a blocking solution for 2 h. Subsequently, cell nuclei were stained with DAPI (Sigma-Aldrich, USA) at a dilution of 1:1000 in the blocking solution for 10 min. The sections were cleaned, placed in glycerol buffer, and examined using an Olympus FV1000 confocal laser scanning microscope. Negative controls were conducted by omitting the primary antisera from the staining. All negative control sections exhibited very weak or absent immunoreactivity. The experiment was conducted in triplicate.

### Image analysis

Immunoreactivity of MUCs-like-ir in the FG, MG, and HG was observed and captured using an Olympus laser-scanning confocal microscope (Olympus America in Center Valley, PA, USA). Imaging and data processing were conducted according to the description provided (Vetkama et al. 2024). Sequential scans were conducted on digestive tissues for each fluorophore to generate individual images for each label, as well as an overlay image of the three to four channels for each optical slice. After acquiring projections of imaging stacks, consecutive optical sections were combined into a unified image plane. The projected pictures were created using subsets of the z-stacks. The thickness of the optical sections in a confocal stack was tuned and maintained consistently throughout a preparation. The various laser intensities provided by Alexa Fluor 488 filter sets, Alexa Fluor 568 filter sets, Alexa Fluor 647 filter sets, DAPI filter sets, and image-processing software were used. The photos were produced from the Olympus confocal system as TIFF files and then transferred to Adobe Photoshop software for adjusting brightness and contrast as needed to achieve optimal clarity. Negative controls for each fluorochrome were scanned using the same parameter settings as those used in immunolabelling with MUCs.

### Assessing the intensities and thickness of MUC-like-ir in the digestive organs during ovarian stages

The relative intensities of MUCs-like-ir in the digestive organs during the ovarian cycle were quantified using ImageJ software (NIH, Bethesda, MD, USA, available online at http://rsb.info.nih.gov/ij/), following a previously established method (Vetkama et al. 2024), while the thickness of MUCs-like-ir layers was measured based on the description (Liu et al. 2020), with some modifications. Digital photographs of the sections at 20X and 40X magnifications were captured from a minimum of five randomly selected sections in each area for analysis. The resolutions and pixels of photos were specified as described, and the images were thereafter converted to grayscale. The ImageJ software's removed background function was utilized to reduce any background signal. The intensities were measured three times. However, we controlled key factors, such as immunohistochemical procedures, timing of incubation with primary and secondary antisera, adjustment of laser channels to ensure accurate measurement of MUCs-like-ir intensities in each stage and minimize potential bias.

### Statistical analyses

Data were presented as means ± S.E.M. The data were then analyzed for statistical differences with the SPSS program from Windows software (SPSS Inc., Chicago, IL, USA), using a one-way analysis of variance (ANOVA) and Tukey post hoc test. A probability value less than 0.05 (*P* < 0.05) indicated a significant difference.

## Results

### Morphological and histological structures of the foregut (FG), midgut (MG), and hindgut (HG) in female *M. rosenbergii*

**Fig. 1:**
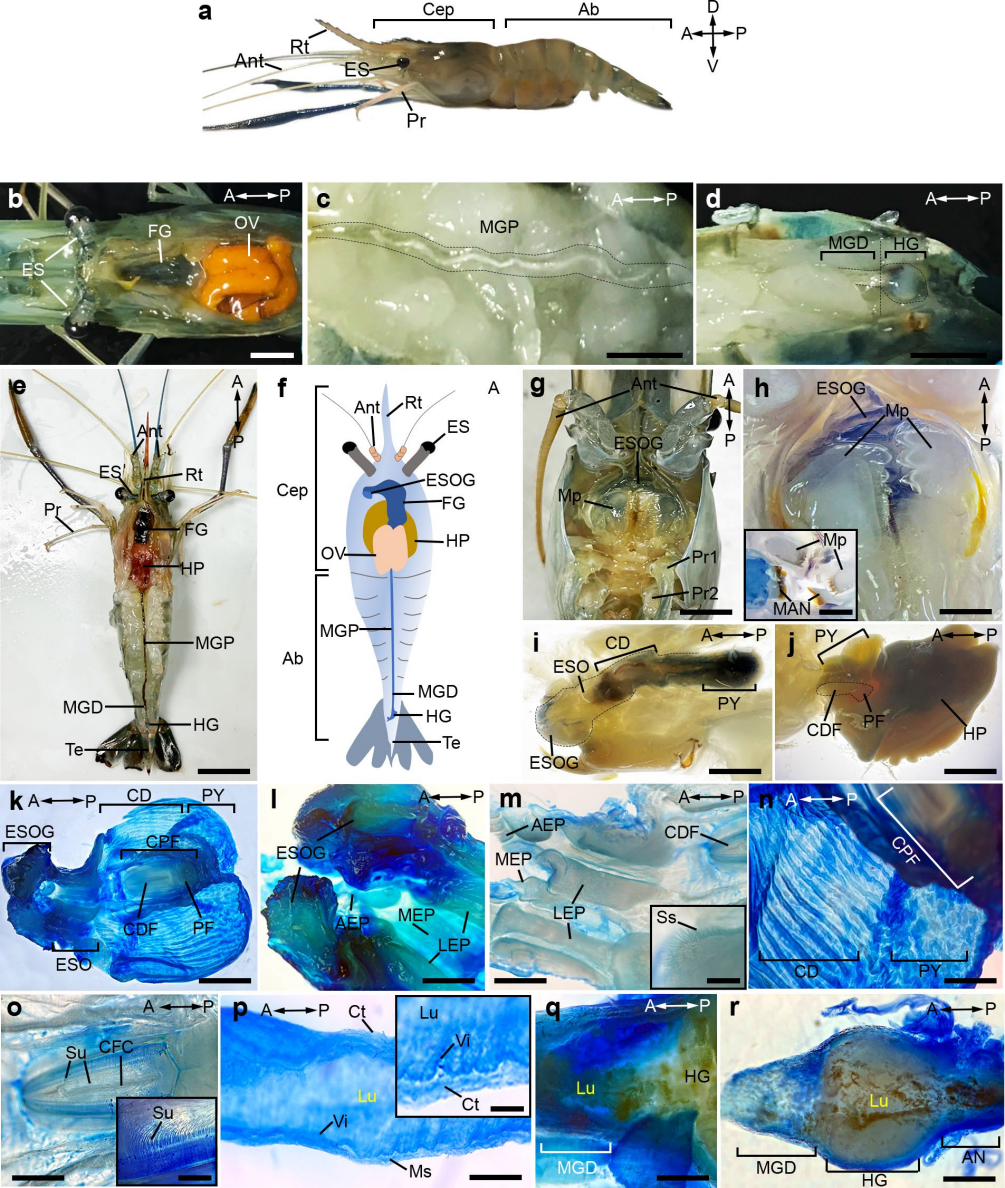
Gross anatomy, and histological images of female prawns, *M. rosenbergii*. **a)** External morphology of female, from the anterior to posterior axes, containing the Cep and Ab regions. **b-e)** Top view of the organization of digestive organs, showing the location of the FG, HP (beneath OV) at the Cep region, and the MGP, MGD, and HG at the Ab region. **f)** A schematic diagram illustrating the digestive organ organization. **g-h)** The ventral view displays the mount opening and its associated structures, which include the mandibles, two Mps, and the ESOG structures **(h)**. Dissected mount part demonstrating a pair of Mop (molar process) (inset of **h**). **i)** The top view of the Cep regions reveals the dissected ESOG, ESO, CD, and PY parts. **j)** On the anterior part of HP, the CDF and PF are located dorsally. **k-n)** Staining the dorsal view of the FG opening with methylene blue reveals the organization of numerous longitudinal ridges in the ESO regions, comprising the AEP, MEP, and LEP **(l-m)**. Along the outer ridge (inset of **m**), there are several Ss. **n)** A representative image showing longitudinal ridges in the CD and PY regions. **o)** The CDF contains rows of the long Su (inset of **o**). **p-r)** Dissected MGP **(p),** MGD **(q),** HG **(r),** and AN **(r)**. A, anterior; Ab, abdomen; AEP, anterior esophageal plate; AN, anus; Ant, antennae; CD, cardiac; Cep, cephalothorax; CPF, cardio-pyloric filter; CDF cardiac filter; CFC, cardiac filter center; D, dorsal; ES, eye stalk; ESO, esophagus; FG, foregut; HG, hindgut; HP, hepatopancreases; LEP, lateral esophageal plate; Lu, intestinal lumen; MEP, medial esophageal plate; MG, midgut; MGD, distal part of midgut; MGP, proximal part of midgut; Mop, molar process; Mp, mouthpad; Ms, muscle; OV, ovary; P, posterior; Pc, procuticle; PF, pyloric filter; Pr, periopod; Pr1, periopods paired 1; Pr2, periopods paired 2; PY, pylorus; Rt, rostrum; Ss, simple setae; Su, serrulate setae; Te, telson; V, ventral; Vi, villi. Scale bar 1 cm. (**a-d**), 2.5 cm (**e–g**), 0.5 cm (**h-j**), 300 µm (**k**), 200 µm (**l-p, r**), 100 µm (**q**), 50 µm (inset of **o, p**)

The FG is situated in the cephalothorax area (Cep), while the MG and HG were observed in the dorsal region of the abdomen (Ab) (Fig. 1a-d). The FG consists of the ESO, CD, and PY (Fig. 1e, f, i, j, and k). The pair of proximal endopods of the maxillipeds are located in front of the mandibles in the ventral view of the Cep area (Fig. 1g). The mandibles exhibit a spindle-shaped form and consist of teeth and a molar located in the mouth (Mo) (Fig. 1h). The ESOG was observed beneath the ESO, and its borders were delineated by the pair of maxillipeds and the molar process (Fig. 1g,h). The ESOG has a triangular shape comprising transplanted colors covered by a rigid epicuticle (Ec) (Fig. 1k,l). The ESO is a compact cylindrical structure, measuring approximately 0.3–0.5 cm in length, that extends from the Mo and subsequently connects to the CD part (Fig. 1i). The ESO ossicles consist of three components: the anterior esophageal plate (AEP), the middle esophageal plate (MEP), and the lateral esophageal plate (LEP) (Fig. 1l). The ventroanterior region of the ESO contains the AEP, while the ventrolateral region houses the pair of LEP. Nevertheless, the MEP was observed at the ventroposterior region of the ESO (Fig. 1m). The ESO ossicles contained simple setae, as shown (Fig. 1m).

We identified a sac of the CD part in the central region of the FG, and the CD wall displays numerous elongated ridges (Fig. 1n). The middle region of the CD floor houses the cardiac filter (CDF), while the apical surface of the CDF displays the serrulate setae (Fig. 1j,o). The PY is located in the posterior region of the CD and contains many longitudinal ridges. The PY folds are higher than those of the CD section (Fig. 1j, k, n). The MG part is a linear, cylindrical formation with a length of about 3.5–4 cm. It continues from PY in the antero-dorsal region, and extends towards the posterodorsal area of the Ab section (Fig. 1e, g). The MG is anatomically separated into two parts: the proximal part (MGP) and the distal part (MGD); the MGD is located close to the HG (Fig. 1p, q). We observed longitudinal folds and a layer of connective tissues (Ct) around the MG (Fig. 1p). The HG begins in the posterodorsal region and continues to the anus (AN). The HG is distinguished by its spherical morphology and pliable gelatinous content, encompassed by a protective Ct layer. In addition, a longitudinal fold was detected in the HG, which stretches throughout the middle lumen of the HG (Fig. 1r).

### Histology and histochemical characteristics of the FG in female *M. rosenbergii*

**Fig. 2:**
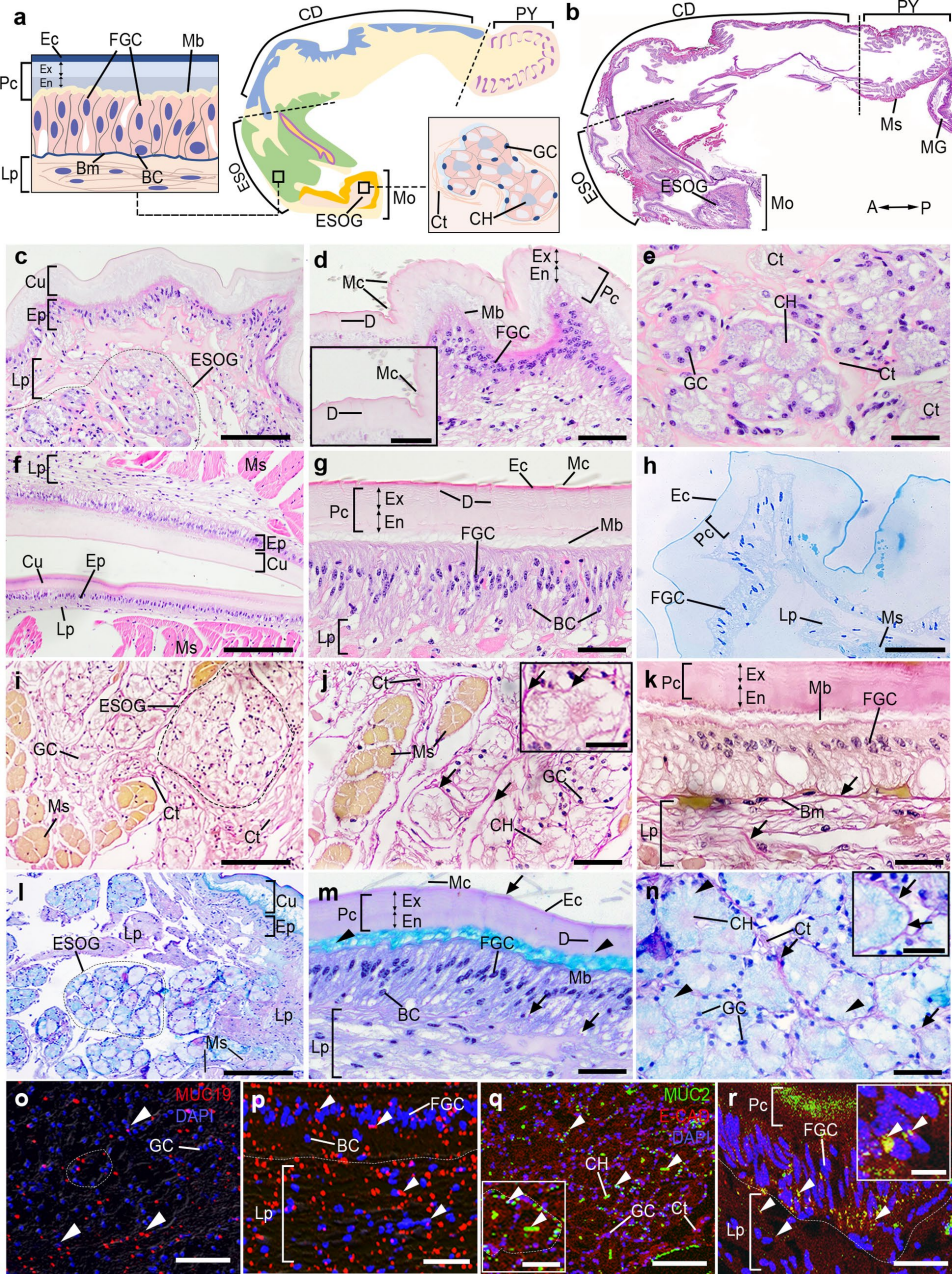
Histological structures and histochemical characteristics of the ESOG and ESO parts in female prawns, *M. rosenbergii*. **a)** Schematic diagrams of the ESO and ESOG organizations. **b)** A low magnification image demonstrating the location of the ESOG and ESO, with the orientation given on the bottom right. **c-g)** Images showing details of the ESOG **(c-e)**, and ESO **(f-g)**. **c, f)** Low magnification images demonstrating the Cu, Ep, Lp, and Ms layers of the ESOG and ESO. **d, g)** The ESOG and ESO exhibit the FGCs, and BCs located at the basal part of the Ep layer. The Ec, Pc, and Mb parts of the Cu layer were observed, and the Pc consists of the Ex and the En layers. The D and Mc structures were present in the Pc, and Ec layers, respectively (inset of **d**). A high-magnification image shows the ESOG, in which the GCs are rosettes-like structures **(e)**. **h)** Semithin section through the ESO. **i-k)** The reaction of Verhoeff's Van Gieson (VVG) in the ESOG **(i-j)**, and ESO **(k)**. In both the ESOG and ESO parts **(j,k)**, collagen fibers (black arrows) were visible**. l-n)** The reactivity of PAS/AB in the ESOG **(l**,**n)**, and ESO **(m)**. Collagen fibers exhibited a positive PAS reaction, as indicated by the black arrows. The strong positive AB reaction (black arrowheads) was present in the GCs of ESOG **(n)**, and the Mb layers of ESO **(m)**, while a strong PAS color was observed in the CH and the basal surface of the GC lobules. **o-p)** The MUC19-like-ir (red) was present in the GCs of ESOG and ESO, and the nuclei were counterstained with DAPI (blue). The pictures “**o-p**” are merged with DIC images. An intense MUC19-like-ir was detected in the GCs (**o**, white arrowheads), FGCs, Lp layers (**p**, white arrowheads). **q-r)** MUC2-like-ir (green), and E-CAD-ir (red) in the ESOG and ESO. An intense MUC2-like-ir was detected in the GCs, CH, Ct (**q**, white arrowheads), FGC, Pc, and Lp layers (**r,** white arrowheads). A, anterior; BC, basal cell; Bm, basement membrane; CD, cardiac; CH, central channel; Ct, connective tissue; Cu, cuticle; D, duct; Ec, epicuticle; ESO, esophagus; ESOG, esophageal gland; Ep, epithelium; En, endocuticle; Ex, exocuticle; FGC, foregut cell; GC, gland cell; Lp, laminar propria; Mc, microspine; Mb, membranous; MG, midgut; Mo mouth; Ms, muscle; P, posterior; Pc, procuticle; PY, pylorus. Scale bars 300 μm (**b**), 100 μm (**c, f, i, l, o, q**), 50 μm (**d, e, g, h, j, k, m, n, p, r**),10 μm (inset of **d, j, q**)

The Mo part consists of three distinct layers: the cuticle (Cu), the epithelium (Ep), and the lamina propria (Lp) (Fig. 2a-c). The Ep nuclei are predominantly spherical in shape. The Cu has three layers: the outermost layer is called the epicuticle (Ec), the middle layer is the procuticle (Pc), which further separates into the exocuticle (Ex) and endocuticle (En), and the innermost layer known as the membranous layer (Mb) (Fig. 2a,c,d,h). The Mb is the thin layer that covers the Ep's apical surface. By employing traditional histological analysis, we categorized the cell types of the Ep of the ESO according to the distinctive features and positioning of the cells that line the basement membrane (Bm) (Fig. 2a,c,d,h). The Ep of the Mo is composed of two distinct cell types: foregut cells (FGCs), characterized by a pseudostratified columnar epithelium, and basal cells (BCs), which have round-shaped nuclei positioned near the Lp layer (Fig. 2c,d).

The Ep of the ESO is similar to that of the Mo part. We observed a significant number of gland cells (GCs) beneath the Mo section (Fig. 2c, e), and the Ct separates the GC into lobules. Every GC exhibits a pyramidal structure and encloses a central channel (CH), similar to acinar structures. Glands in female *M. rosenbergii* demonstrated histological characteristics of multicellular glands (Fig. 2e,i,j). The cytoplasm of GCs contains the remaining mucous secretion, with the nuclei of these cells located at the basal area (Fig. 2e). The FGCs and BCs were observed in the Ep layers, while the Ct exhibited a spongy-like shape in the Lp layer (Fig. 2f,g). The Lp is a loose connective tissue layer forming a deeper part of the mucosal layer of the FG mucosa. The Ms layer is thick and lies beneath the Ep layer (Fig. 2f). The Verhoeff-Van Gieson staining (VGG) revealed abundant collagen fibers stained magenta, while distinct Ms bundles were stained in yellow. However, we were unable to identify any elastic fibers in the aforementioned digestive region (Fig. 2i,j,k). The reactions of PAS and AB varied across different regions of the FG. Within the ESO, the Cu layer exhibited a strong PAS staining, whilst the Pc layer displayed a moderate level of PAS staining. Nevertheless, the Mb layer of the Cu exhibited strong AB staining, indicating the presence of glycoproteins (Fig. 2l,m). The ESOG showed intense AB staining in the acinus-like structure of GCs (Fig. 2l,n). The Ec and Ct surrounding the lobules demonstrated a strong positive staining of PAS (Fig. 2m,n). Both MUC19-like-immunoreactivity (-ir) and MUC2-like-ir were detected in the GCs of the ESOG and the Lp layers of the ESO (Fig. 2o-r). However, a strong MUC-like-ir was observed on the upper surface of FGCs (Fig. 2p,r).

**Fig. 3:**
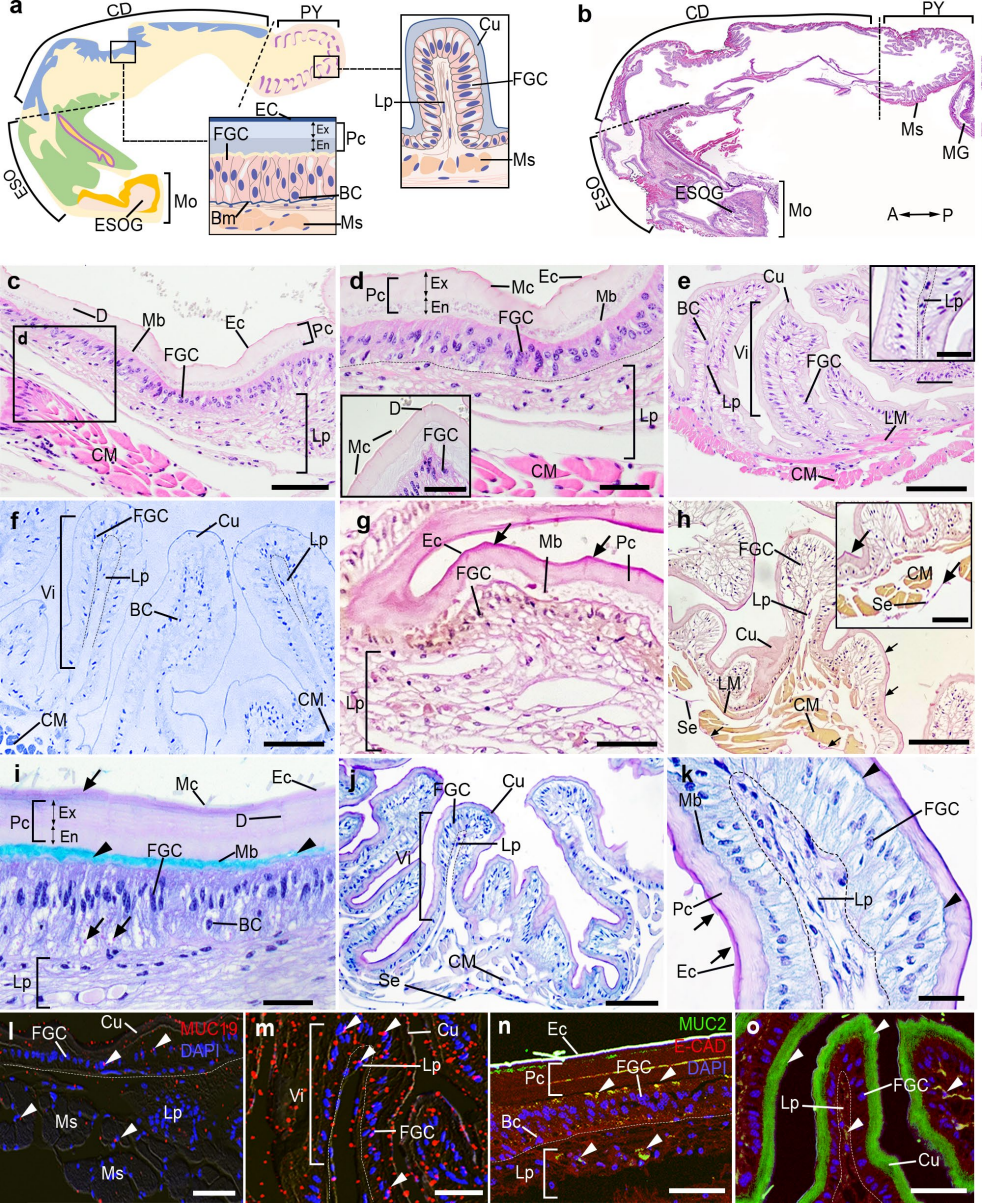
Histological structures and histochemical characteristics of the CD and PY parts in female prawns, *M. rosenbergii*. **a)** Schematic drawings of the CD and PY organizations. **b)** The location of the CD and PY regions, with their orientation, is given on the bottom right. **c)** The CD section displays the Cu, Ep, Lp, and Ms layers. **d)** A high magnification image taken from **c** exhibiting the Ec, Pc, Mb, and FGC, in which the Mc, and D are present in the Cu layer (inset of **d**). **e–f)** The PY part by H&E **(e)**, and the semithin section **(f)**, showing the Vi structure, and Lp layer at the core of Vi (inset of **e**). **g-h)** The VVG staining of the CD **(g)**, and PY parts **(h)**. **i-k)** The PAS/AB staining of the CD **(i),** and PY parts **(j-k)**. The strong reaction of PAS was seen in the Ec, CD, and PY **(i, k** black arrows). The strong AB reactivity was seen in the Mb layer of CD (**i**, black arrowheads). **l-m)** MUC19-like-ir (red) in the CD, and PY, with nuclei counterstained with DAPI (blue). The pictures “**l-m**” are merged with DIC images. MUC19-like-ir was localized in the Cu, FGCs, and Lp (**l-m,** white arrowheads). **n–o)** MUC2-like-ir (green), and E-CAD-ir (red) in the CD and PY. An intense MUC2-like-ir was observed in the Cu and FGCs of the CD part (**n,** white arrowheads), while strong MUC2-like-ir was detected in the Cu and Lp of the PY part (**o,** white arrowheads). A, anterior; BC, basal cell CD, cardiac; CM, circular muscle; Cu, cuticle; D, duct; Ec, epicuticle; En, endocuticle; Ep, epithelium; Ex, exocuticle; FGC, foregut cell; Lp laminar propria; LM, longitudinal muscle; Mb, membranous; Mc, microspine; MG, midgut; Mo mouth; Ms, muscle; Pc, procuticle; PY, pylorus; Se, serosa; Vi, villi. Scale bars 300 μm (**b**), 100 μm (**c, e, f, h, j**), 50 μm (**d, g, i, k, l, m, n, o,** inset of **d, e, h**)

The CD part is composed of the Cu, Ep, Lp, and thick circular (CM) and longitudinal muscle (LM) layers (Fig. 3a-c). The CD is encased with layers of Ep coated with a layer of Cu. The Cu lines small finger-like projections called villi (Vi). The FGCs in the small Vi are characterized by a straightforward transition from simple columnar to the pseudostratified columnar epithelium (Fig. 3d). In the CD portion, FGCs form a lining along the Vi, Cu, Lp, and Ms layers. There are layers beneath the Ep, including the Lp situated underneath the Ep, the CM layer, and the serosa in the outermost layer (Fig. 3c,d). The collagen fibers were extensively spread across the Lp layer, while no elastic fibers were detected in the CD region (Fig. 3g). The Cu layer of the CD showed a strong PAS staining, while the Pc layer exhibited a moderate level of PAS staining. However, the Mb layer on the upper surface of FGCs exhibited intense AB staining (Fig. 3i). The PY is lined by a simple columnar epithelium bearing a thinner Cu layer compared to that of the CD. The PY is characterized by many longitudinal ridges and Vi, and is surrounded by an inner layer of oblique-oriented LM and an outside layer of CMs. The two cell types, including FGCs and BCs, were observed (Fig. 3,e,f). Additionally, serosa (Se) consists of a layer of squamous cells and a delicate layer of loose connective tissue. Se is located in the outermost layer of digestive organs (Fig. 3h,j). However, we found many collagen fibers in the Lp layer, but this digestive region showed a notable absence of elastic fibers (Fig. 3h). The expression of AB-positivity was strongly observed in the Mb part of Cu, while strongly PAS-positive staining was seen in the Ec part of Cu (Fig. 3j,k). AB staining was found in Mb, while PAS staining was also observed in the Ct and Ec of the Cu layer (Fig. 3j,k). The apical region of FGCs, Lp, and Cu layers of CD and PY parts showed the presence of both MUC19- and MUC2-like-ir (Fig. 3l-o). Additionally, strong MUCs-like-ir was observed on the FGCs and their upper surface (Fig. 3m-o).

### Histology and histochemical characteristics of the MG in female *M. rosenbergii*

**Fig. 4:**
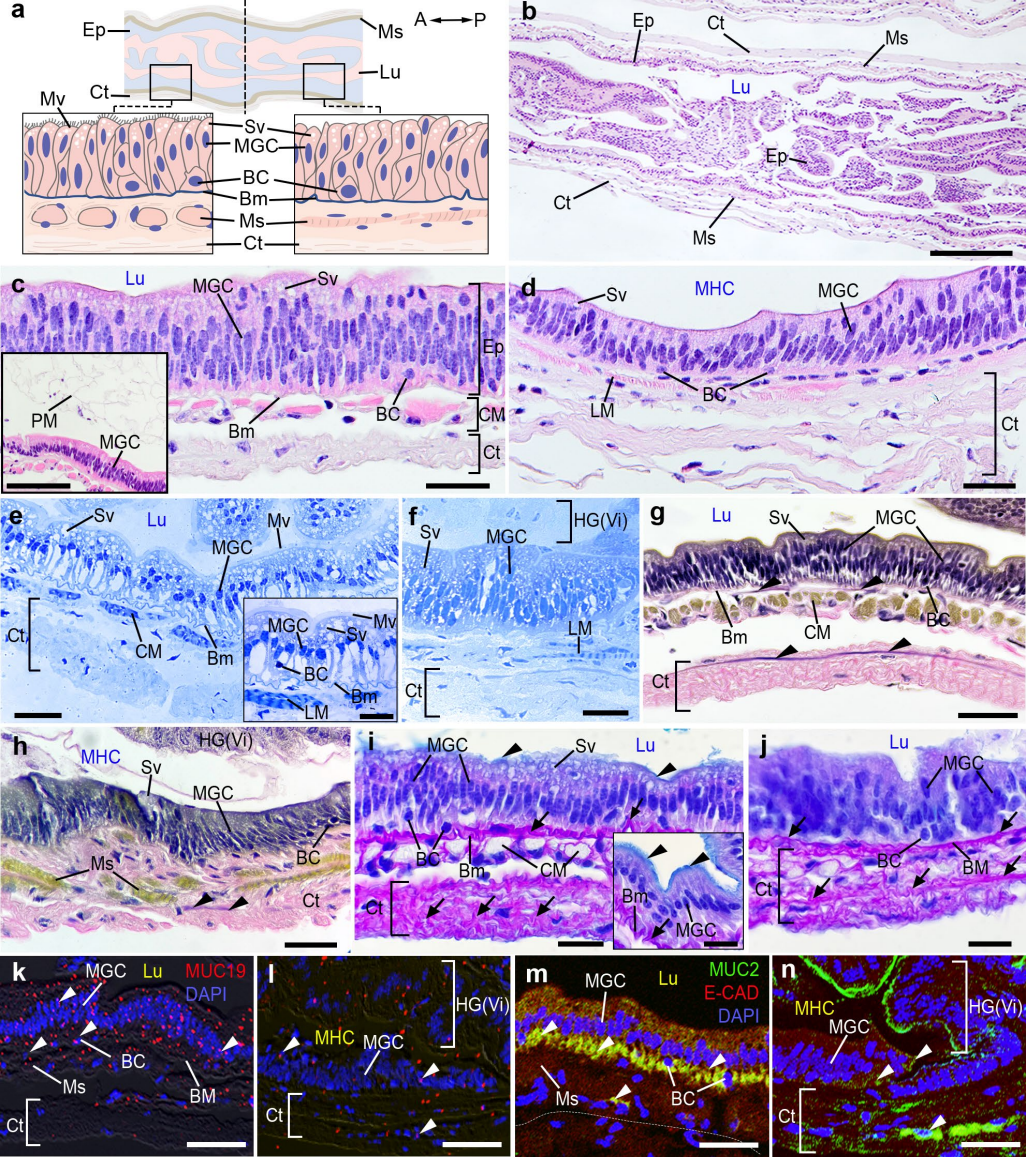
Histological structures and histochemical characteristics of the MG in female prawns, *M. rosenbergii*. **a)** A schematic representation of the MG with the orientation is given on the top right. **b)** The longitudinal section of the MG, stained with H&E, displays the Ep, Ct, Ms, and Lu layers. **c-d)** The MGP **(c)** and MGD **(d)** by H&E show the MGCs lining on the Bm, and Sv at the apical surface of MGCs. The PM was present at the MGP Lu (inset of **c**). **e–f)** Semithin section of MGP **(e),** and MGD **(f)** exhibiting the MGCs, Mv, and a distinct Bm. **g-h)** The reaction of VVG in the MGP **(g),** MGD **(h).** The elastic fibers were present at the Bm, and Ct layers, at the MGP and MGD (black arrowheads). In the MGP, there are bundles of collagen fibers, with dense strips of elastic fibers in place of the outer LM layer **(g-h)**. **i-j)** The reactions of PAS/AB in the MGP **(i),** and MGD **(j)**. A strong AB reaction was observed at the Mv of MGCs (black arrowheads), while a strong reaction of PAS was detected in the Bm and Ct layers (black arrows). **k-l)** MUC19-like-ir was detected in the MGP, and MGD, with nuclei counterstained with DAPI (blue). The pictures “**k-l**” are merged with DIC images. Intense MUC19-like-ir was seen in several cells, including the MGCs and Ct layers (**k, l,** white arrowheads). **m–n)** MUC2-like-ir (green), and E-CAD-ir (red) were seen in the MGCs, the Ms layer of the MGP part (**m, n,** white arrowheads). A, anterior; BC, basal cell; Bm, basement membrane; Ct, connective tissue; CM, circular muscle; Ep, epithelium; HG(Vi), hindgut villi; LM, longitudinal muscle; Lu, intestinal lumen; MGC, midgut cell; MGD, midgut distal; MHC, midgut-hindgut channel; MGP, midgut proximal; Ms, muscle; Mv, microvilli; P, posterior; PM, peritrophic membrane; Sv, subapical vacuoles. Scale bars 200 μm (**b**), 100 μm (inset of **c**), 50 μm (**c-n**), 10 μm (inset of **e**,** i**)

The MG is divided into two main parts: the proximal part (MGP) and the distal part of the MG (MGD). The Ep of the MGP is composed of a pseudostratified columnar epithelium (Fig. 4a-c). The cellular composition of MG primarily consists of two types: midgut cells (MGCs) and BCs, which are located above the prominent Bm (Fig. 4a-c). The MGCs in the MGP part consist of cells with weakly basophilic and homogeneous cytoplasm, containing round nuclei (Fig. 4c,e). When observed in H and E and semithin sections, the MGCs have Mv on their apical surface and the upper zone of the cytoplasm in these cells has abundant subapical vacuoles (Fig. 4c,e). Additionally, a conspicuous basal membrane (BM) connects with the MGCs' basal surfaces. Nevertheless, the MG epithelium lacks the Cu layer. The CM layer was prominently formed and positioned beneath the Bm layer of the Ep layer (Fig. 4c,e,g). Additionally, the outermost layer of the MGP comprises compact bands of collagen fibers interlaced with dense strips of elastic fibers (Fig. 4g).

In the MGD, the BCs exhibited round nuclei, which are visible in the basal portion of the Ep layer, as demonstrated in the MGP (Fig. 4d,f). The cytoplasm of cells showed moderate and homogeneous PAS staining. Notably, the Ct that enveloped the Ms and Ct of the MGP prominently displayed elastic fibers shown in black (Fig. 4h). However, the obliquely oriented LM were arranged unevenly in the Ct layer of MGD (Fig. 4h). Strong AB-positivity was observed on the Mv that coats the external surface of the Ep of the MGCs. Nevertheless, a strong PAS reaction was observed in the Bm and Ct layers (Fig. 4i). In the MGD, the Bm and Ct layers showed a strong PAS reaction, while only a faint AB reaction was seen (Fig. 4j). Both MUC19- and MUC2-like-ir was observed on the apical surface of both the MGCs (Fig. 4k,m). However, the expression of both MUCs-like ir was decreased in the Ms and Ct layers of the MGP (Fig. 4k,m), as well as in the MGD parts (Fig. 4l,n). Additionally, weak MUC-2-like-ir was observed in the MGCs and Ct layer of the MGD region (Fig. 4l,n).

### Histology and histochemical characteristics of the HG in female *M. rosenbergii*

**Fig. 5:**
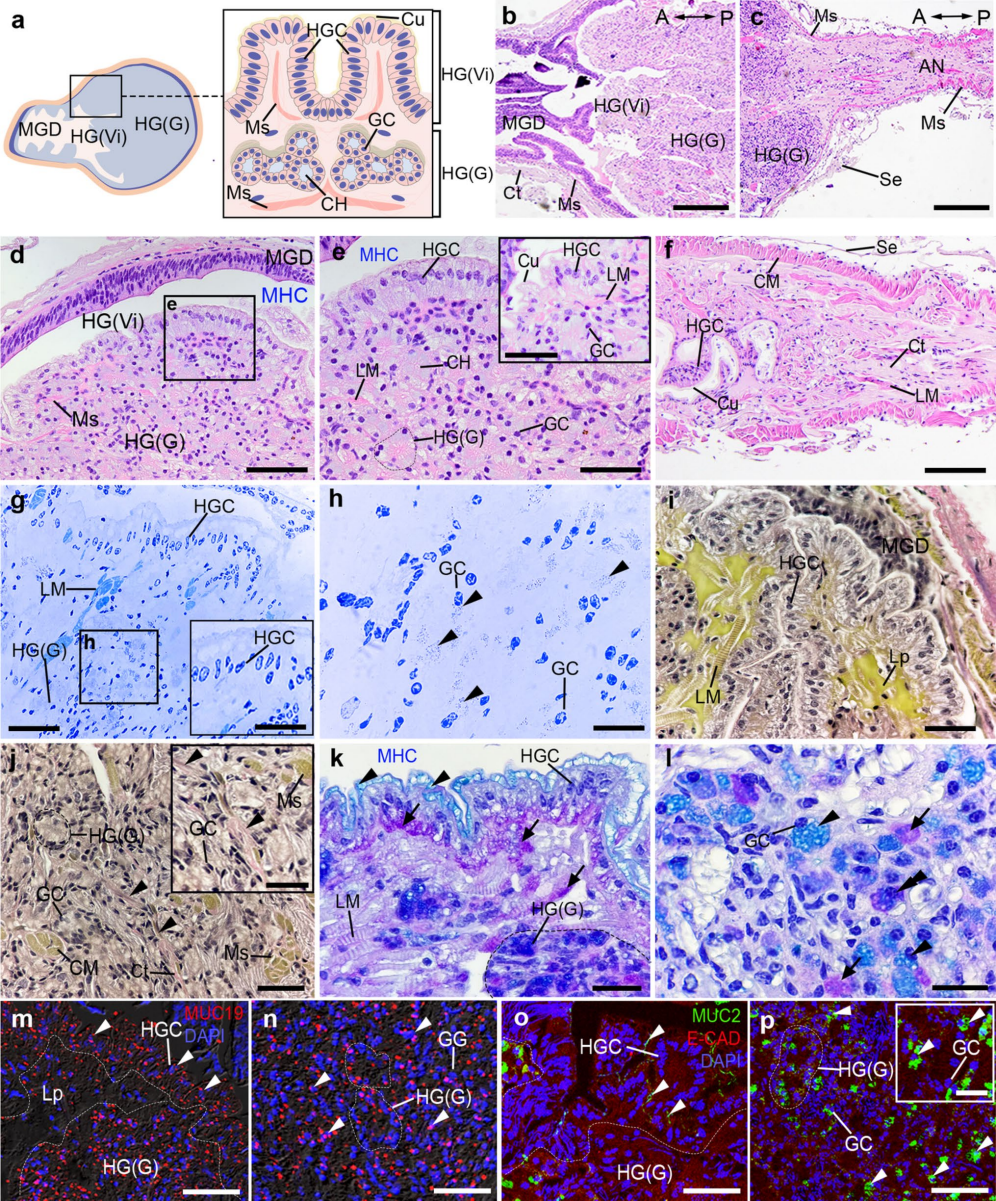
Histological structures and histochemical characteristics of the HG in female prawns, *M. rosenbergii*. **a)** A schematic representation illustrating the details of the HG. **b-c)** Longitudinal sections of the HG by H&E exhibiting the MGD, HG(Vi), HG(G) **(b),** and the AN parts **(c),** with the orientation given on the top right. **d)** Image showing the HG(Vi) and HG(G) part, as well as the MHC, which is a space between MG and HG. **e)** Higher magnification image taken from **d** exhibiting HGCs lining the Vi at the HG(Vi) and GCs at HG(G), and showing the thin Cu layer on the apical surface of the HGCs (inset of **e**). **f)** At the HG-AN junction, the circular Ms becomes increasingly thicker in the anal region. **g-h)** Semithin images of the HG exhibiting detail of HG(Vi), and HG(G). Images taken from g show several granules within GCs' cytoplasm (**h**, black arrowheads). The histological images from **i** to **j** demonstrate the reaction of VGG in the HG(Vi) **(i)** and HG(G) **(j).** The elastic fibers were observed in the Ct, which surrounds the GCs of HG(G) (**j,** black arrowheads). **k-l)** Images showing the reaction of PAS/AB in the HG(Vi) **(k)** and HG(G) **(l)**. Strong AB (black arrowheads), and PAS (black arrows) reactions were detected in both HGCs of HG(Vi) and GCs of HG(G). The mixed AB/PAS reaction was also seen in the GCs of HG(G) (**l,** double black arrowheads). **m–n)** The expression of MUC19 (red)-like-ir in the HG(Vi), and HG(G), with nuclei counterstained with DAPI (blue). The pictures “**m–n**” are merged with DIC images. MUC19-like- ir was observed in the HGCs, GCs, and Lp layers (**m, n,** white arrowheads). **o-p)** MUC2-like-ir (green) and E-CAD-ir (red) were seen in HG(Vi), and HG(G). Strong MUC2-ir was detected in the HGCs of HG(Vi) part (**o**, white arrowheads), and the GCs of HG(G) (**p,** white arrowheads). A, anterior; AN, anus; Ct, connective tissue; CM, circular muscle; Cu, cuticle; Ep, epithelium; GC, gland cell; HGC, hindgut cell; HG(Vi), hindgut villi; HG(G), hindgut gland; HGC, hindgut cell; LM, longitudinal muscle; MHC, midgut-hindgut channel; Ms, muscle; P, posterior; Se, serosa. Scale bars 200 μm (**b, c**), 100 μm (**d, f**), 50 μm (**e, g-h, i-p**), 10 μm (inset of **e, g, i, p**)

The anterior part of the HG is enveloped by the MGD, which contains small folds of Vi (Fig. 5a-d). The HG consists of two different parts, namely, the hindgut villi (HGVi) and the hindgut gland (HGG) (Fig. 5d,e). The cell types of the HG comprise the hindgut cells (HGCs) located in the HGVi, while the gland cells (GCs) are situated in the HGG region. The HGCs are lined by a simple columnar epithelium with a thin Cu layer (Fig. 5d,e). We observed the inner slender fibers of LM in the HGVi and HGG, and the outer thin CM layer (Fig. 5e, f). However, the thick CM layer was found in the HG-AN junction and AN part (Fig. 5e, f). Using semithin sectioning and VVG staining, we observed a widespread occurrence of a large number of small granules present in the GCs (Fig. 5g,h), and individual LM fibers projecting just beneath the Ep cells and extending toward the Vi. In addition, the HGG is surrounded by a combination of collagen fibers and elastic fibers (Fig. 5i,j). However, the elastic fibers were rarely present in the HGVi (Fig. 5i). In the HGVi, the AB reaction was evident on the apical surface of HGCs, while the basal portion of HGCs exhibited intense PAS staining (Fig. 5k). Using the PAS/AB staining, we identified three distinct HGG cell types, based on their staining properties. These cell types include the acid mucous type, the neutral mucous type, and the mixed mucous type. In the acid mucous type, there was a strong expression of the AB reactivity (Fig. 5l), while the neutral mucous cells displayed strong PAS reactivity (Fig. 5l). Nevertheless, a strong reaction of both PAS and AB was observed in the mixed cell population (Fig. 5l). The HGCs of the HGVi sections exhibited both MUCs-like-ir (Fig. 5m,o). Additionally, there was a strong expression of both MUC19- and MUC2-like-ir in the GCs of the HGG part (Fig. 5n,p).

### Changes in the histological and histochemical characteristics in the FG, MG, and HG during the ovarian cycle

**Fig. 6:**
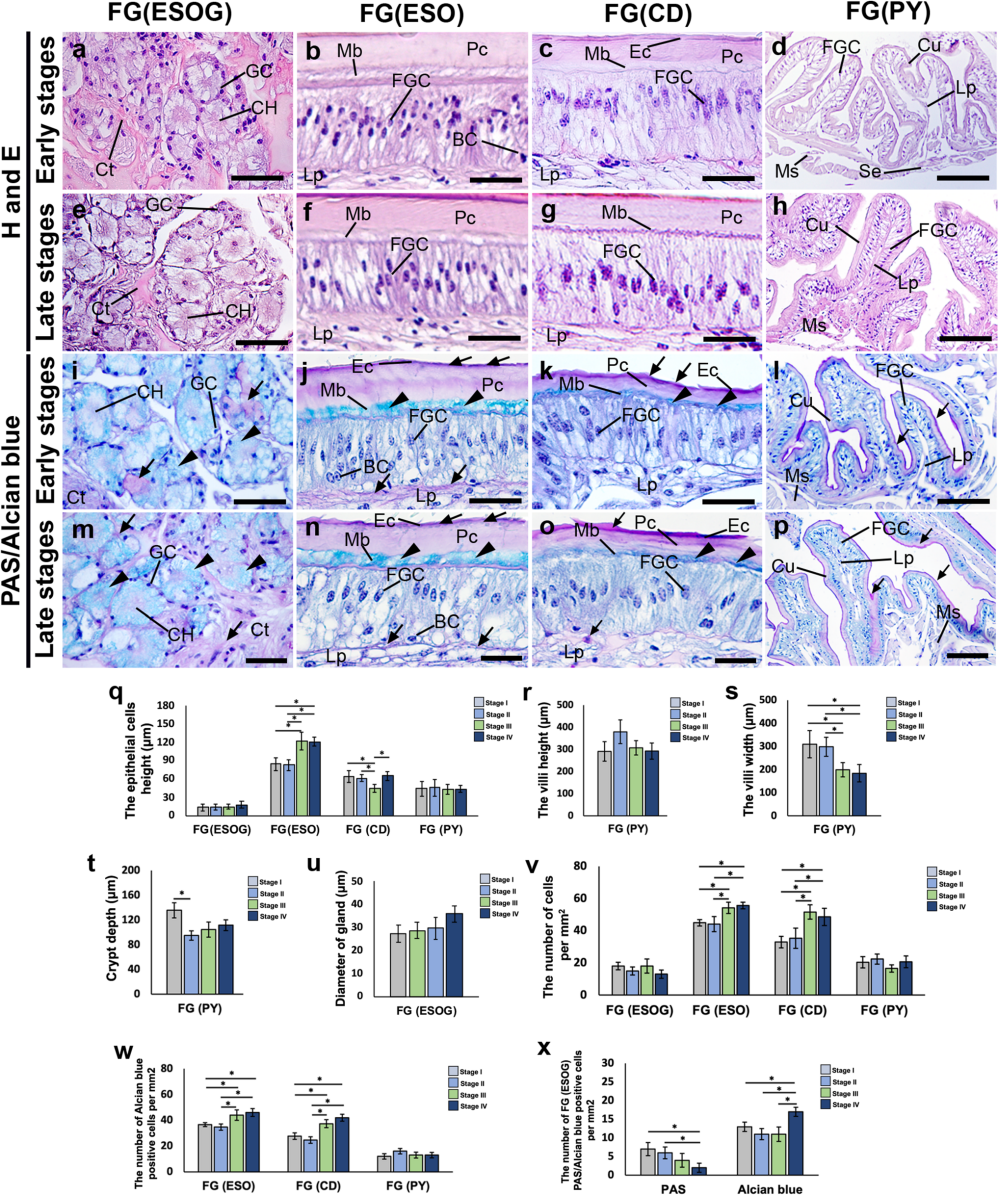
Changes in histological and histochemical characteristics of the FG structures of female *M. rosenbergii* during the ovarian cycle. **a-h)** Histological structures of the ESOG, ESO, CD, and PY parts during the early **(a-d)** and late stages **(e–h),** respectively. **i-p)** Representative images showing the changes in the PAS reaction (white arrows), and AB (white arrowheads) in the ESOG, ESO, CD, and PY parts during the early **(i-l)** and late stages **(m-p),** respectively. **q)** The epithelial cell height in the ESOG, ESO, CD, and PY parts at different ovarian stages. **r-t)** Changes in the villi height **(r),** villi width **(s),** and crypt depth **(t)** in the PY during ovarian stages. **u)** The diameter of the glands in the ESOG. **v-w)** The number of cells, and the number of positive AB cells in the ESO, CD, and PY parts. **x)** The mean number of positive PAS, and AB cells in ESOG during different ovarian stages. All data are presented as the mean ± SEM. Asterisks indicate significant differences (*P* < 0.05). BC, basal cell; CD, cardiac; CH, central channel; Ct, connective tissue; Cu, cuticle; Ec, epicuticle; ESOG, esophageal gland; ESO, esophagus; FGC, foregut cell; GC, gland cell; Lp, laminar propria; Mb, membranous; Ms, muscle; P, posterior; Pc, procuticle; PY, pylorus; Se, serosa. Scale bars 100 μm (**d, h, l, p**) 50 μm (**a-c, d-g, i-k, m–o**)

**Fig. 7:**
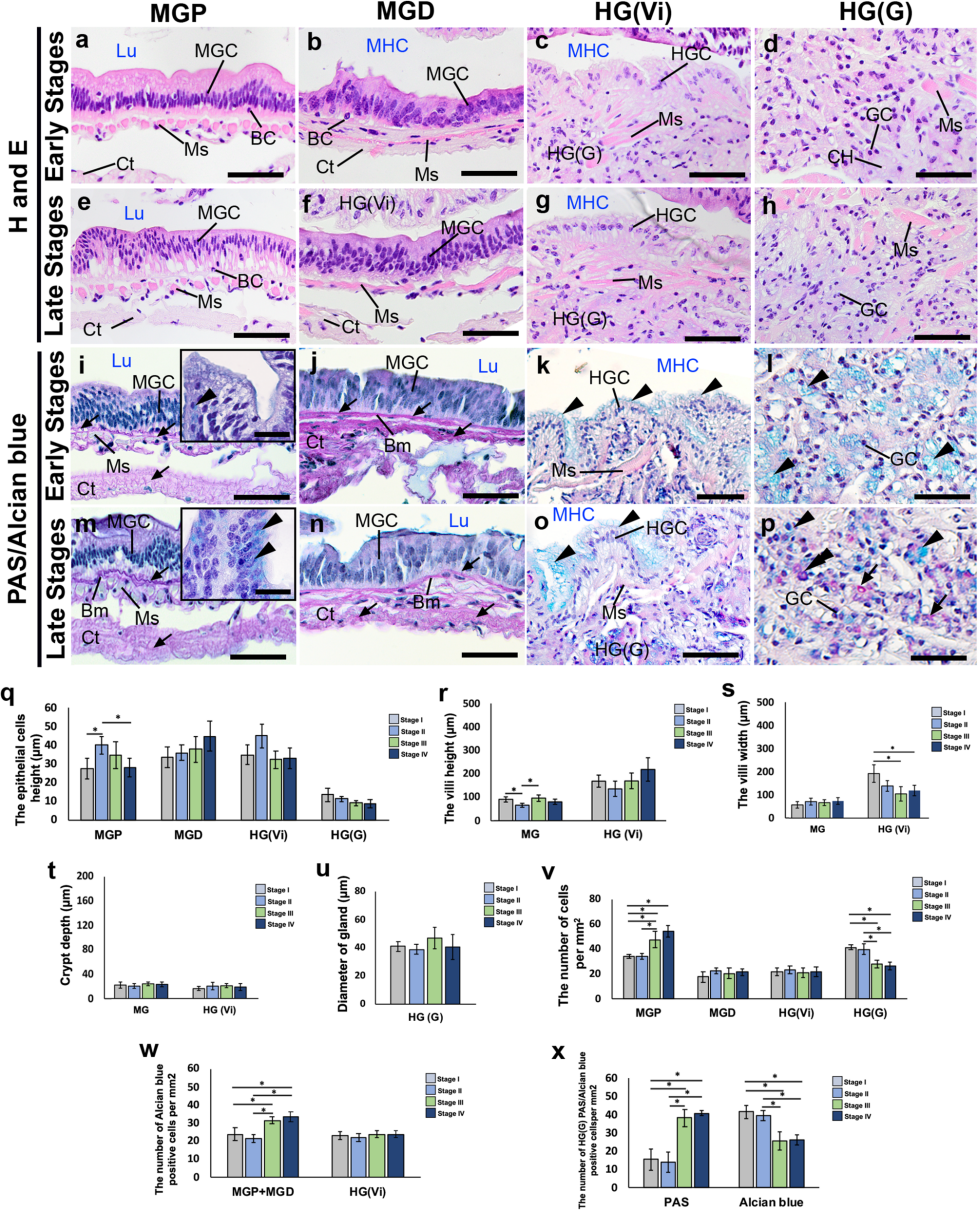
Alterations in histological and histochemical characteristics of the MG and HG structures of female *M. rosenbergii* during the ovarian cycle. **a-h)** Changes in the histological structures of the MGP, MGD, HG(Vi), and HG(G) parts during the early **(a-d)** and late stages **(e–h),** respectively. **i-p)** Representative images showing alterations in the reaction of PAS (white arrows), and AB (white arrowheads) in the MGP, MGD, HG(Vi), and HG(G) sections at the early **(i-l)** and late stages **(m-p),** respectively. **q)** Changes in the epithelial cell height of the MGP, MGD, HG(Vi), and HG(G) during different ovarian stages. **r-t)** Histograms showing changes in the villi height **(r),** villi width **(s),** and crypt depth **(t)** in the MG and HG(Vi). **u)** The diameter of the gland in the HG(G). **v-w)** The number of cells in the MGP, MGD, HG(Vi), and HG(G) parts **(v),** as well as the number of positive AB cells in the MGP and MGD, as well as HG(Vi) **(w)** at ovarian stages. **x)** During ovarian stages, the number of positive PAS and AB cells in the HG (G) was observed. All data are presented as the mean ± SEM. Asterisks indicate significant differences (*P* < 0.05). BC, basal cell; Bm basement membrane; CH, central channel; Ct, connective tissue; GC, gland cell; HGC, hindgut cell; HG(Vi), hindgut villi; HG(G), hindgut gland; MGC, midgut cell; MGD, midgut distal; MGP, midgut proximal; MHC, midgut-hindgut channel; Lu, intestinal lumen; Ms, muscle. Scale bars 50 μm (**a-p**)**,** 10 μm (inset of **i, m**)

The diameter of the ESOG appeared to be slightly larger in the late stages, compared with the early stages (*P* > 0.05) (Fig. 6a,e,u). The height of the Ep cells in the ESO part was significantly greater in the late stages (approximately 120 µm), compared with the early stages (approximately 90 µm) (*P* < 0.05) (Fig. 6b,f,q). On the other hand, the height of the Ep cells in the CD appeared to be higher in the early stages (approximately 68 µm) than in the mid-stage (approximately 47 µm) (*P* < 0.05) (Fig. 6c,g,q). The Ep cell height and villi height of the PY region, appeared to be greater in the early stages, but did not show any statistically significant differences at any stage (*P* > 0.05) (Fig. 6d,h,q,r). Nevertheless, the width of the Vi and the depth of the crypt in the PY were notably greater during the early stages, compared with the late stages (*P* < 0.05) (Fig. 6d,h,s,t). In the late stages, the number of cells in the ESO and CD portions were higher, compared with the early stages (*P* < 0.05) (Fig. 6v). We observed approximately 60 cells in the ESO and approximately 57 cells in the CD during the late stages. In contrast, there were approximately 45 cells in the ESO and approximately 40 cells in the CD during the early stages. This difference was statistically significant (*P* < 0.05) (Fig. 6v). However, there was no significant difference in the cell count within the PY region between the early and late stages (*P* > 0.05) (Fig. 6v). As well, the number of AB-positive cells in the ESO and CD showed a substantial increase in the late stages (approximately 44 cells in the ESO and 40 cells in the CD), compared with the early stages, which had approximately 38 cells in the ESO and 31 cells in the CD (*P* < 0.05) (Fig. 6j,k,n,o,w). The late stages exhibited a substantially higher number of AB-positive cells, compared with the early stages (*P* < 0.05), while the early stages showed a significantly higher number of PAS-positive cells compared to the late stages (*P* < 0.05) (Fig. 6i,m,x). In the MGP, the height of the Ep cells was considerably greater during the early to mid-ovarian stages, but slightly decreased at the late stage (*P* < 0.05) (Fig. 7a,e,q). On the other hand, the height of the Ep cells in the MGD seemed to be higher during the late stages, compared with the early stages (Fig. 7b,f,q). The changes in Ep cell height in HGVi and HGG were greater in the early stages, compared with the late stages, but were not significantly different (*P* > 0.05) (Fig. 7c,d,g,h,q). The height of the small Vi in the MG reached its peak in the mid stage (*P* < 0.05). However, the Vi height in HGVi was higher in the late stages compared with the early stages (Fig. 7r).

The width of the Vi in the HGVi was found to be greater in the early stages, compared with the late stages, although this difference was not statistically significant (*P* > 0.05) (Fig. 7s). No significant difference was observed in the crypt depth of the MG and HGVi. However, the diameter of the HGG appeared to be larger in the late stages than the early stages (*P* > 0.05) (Fig. 7t,u). The number of MGP cells was notably greater in the later stages (about 57 cells), compared with the earlier stages (about 38 cells) (*P* < 0.05) (Fig. 7v). The total cell count of HGG was significantly higher in the early stages, compared with the late stages (*P* < 0.05) (Fig. 7v). The number of positive AB cells in the MGP and MGD was considerably greater in the late stages (about 30 cells), compared to the early stages (about 20 cells) (*P* < 0.05) (Fig. 7i,j,m,n,w). Nevertheless, the number of AB-positive cells in the HGVi section did not show a statistically significant difference (*P* > 0.05) (Fig. 7k,o,w). Notably, the number of PAS-positive cells in the HGG was significantly greater in the late stages (approximately 40 cells), compared with the early stages (approximately 15 cells) (*P* < 0.05) (Fig. 7l,p,x), whereas the number of AB-positive cells was notably higher in the early stages (about 42 cells), compared with the late stages (around 28 cells) (*P* < 0.05) (Fig. 7l,p,x).

### Alterations in the expression of *mucin (MUC)* gene and mucins-like-ir in the FG, MG, and HG during the ovarian cycle

**Fig. 8:**
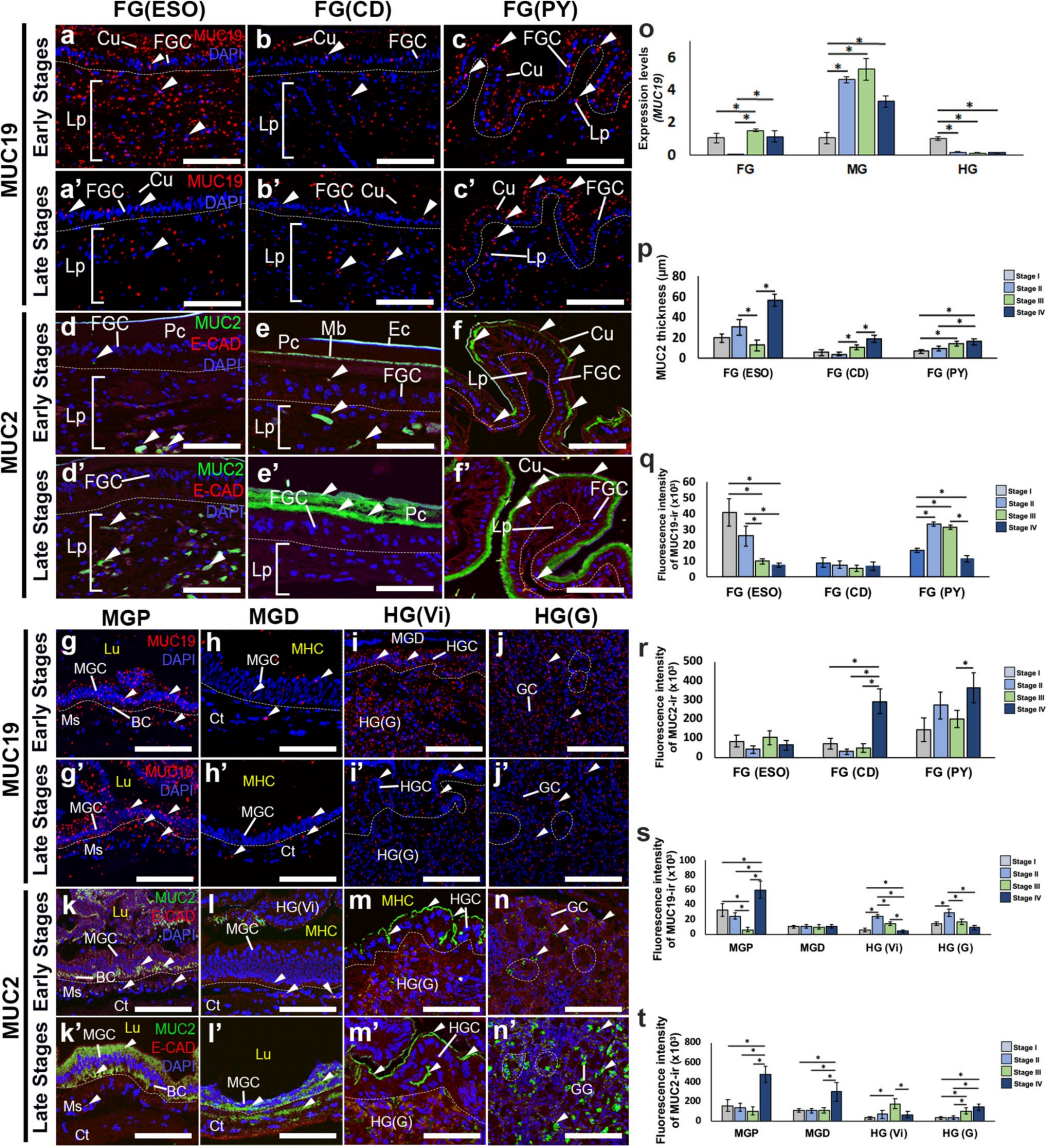
Alterations in the expression of MUC19-like-ir (red), and MUC2-like-ir (green) in the FG, MG, and HG during different ovarian stages of female *M. rosenbergii*, while nuclei were counterstained with DAPI (blue). **a-c, a’-c’)** Changes in the expression of MUC19-like-ir in the ESO, CD, and PY parts at the early (**a-c**), and the late stages (**a’-c’**), respectively. An intense MUC19-like-ir was detected in the Cu, FGCs, and Lp (white arrowheads). **d-f, d’-f’)** MUC2-like-ir in the ESO, CD, and PY parts during the early (**d-f**), and late stages (**d’-f’**). Strong MUC2-like-ir was seen in the Cu, FCG, and Lp (white arrowheads).** g-j, g’-j’)** Representative images exhibiting the expression of MUC19-like-ir in the MGP, MGD, HG(Vi), and HG(G) parts at the early (**g-j**), and the late stages (**g’-j’**). **k-n, k’-n’**) Changes in the expression of MUC2-like-ir in the MGP, MGD, HG(Vi), and HG(G) at the early (**k-n**), and the late stages (**k’-n’**), respectively. **o)** Alterations in the expression of *MUC19* mRNA levels in the FG, MG, and HG during the ovarian cycle, as determined by RT-qPCR. **p-r**) Changes in the MUC2 thickness **(p)** and fluorescence intensity of MUC19-like-ir (**q**) and MUC2-like-ir (**r**) in the ESO, CD, and PY parts during different ovarian stages. **s-t**) Histograms representing the fluorescence intensity MUC19-like-ir (**s**), and MUC2-like-ir (**t**) in the MGP, MGD, HG(Vi), and HG(G) parts during ovarian stages. All data are presented as the mean ± SEM. Asterisks indicate significant differences (*P* < 0.05). BC, basal cell; CD, cardiac; Ct, connective tissue; Cu, cuticle; Ec, epicuticle; ESO, esophagus; FGC, foregut cell; GC, gland cell; HGC, hindgut cell; HG(Vi), hindgut villi; HG(G), hindgut gland; Lp, laminar propria; Lu, intestinal lumen; Mb, membranous; MGC, midgut cell; MGD, midgut distal; MGP, midgut proximal; MHC, midgut-hindgut channel; Ms, muscle; P, posterior; Pc, procuticle; PY, pylorus. Scale bars 100 μm (**a-c, a’-c’, d-f, d’-f’**), 50 μm (**g-j, g’-j’, k-n, k’-n’**)

Alterations in the expression of MUC19-like-ir and MUC2-like-ir were detected in the FG, MG, and HG throughout the ovarian cycles. The presence of MUC19-like-ir was observed in the ESO, CD, and PY regions throughout the early (Fig. 8a-c) and late stages (Fig. 8a’-c’). Based on RT-qPCR assay, the relative expression levels of *MUC19* mRNA of the FG were higher in the late stages, compared to the early stages (*P* < 0.05) (Fig. 8o). Notably, *MUC19* mRNA levels in the MG were significantly higher in the stage II (about 4.2-fold), and peaked at stage III (about fivefold), but the level suddenly declined at stage IV (about 2.7-fold), compared with stage I (*P* < 0.05) (Fig. 8o). However, the expression levels of *MUC19* mRNA in the HG appeared highest at stage I, compared to the late stages (*P* < 0.05) (Fig. 8o). The thickness of MUC2-like-ir in the ESO, CD and PY regions was considerable during the early stages, and reached its peak during the late stages (*P* < 0.05) (Fig. 8p). The fluorescence intensity of MUC19-like-ir was greatest during the early stages, but the lowest intensity was observed during the late stages of the ESO portion (*P* < 0.05) (Fig. 8a,a’,q). In contrast, the intensity of MUC2-like-ir did not show a significant difference between the early and late stages (*P* < 0.05) (Fig. 8d,d’,r). However, there was no significant difference in the intensity of MUC19-like-ir in the CD between the early and late stages (*P* > 0.05) (Fig. 8b,b’,q). The mid-ovarian stage showed the highest intensity of MUC19-like-ir in the PY part, compared with the early stage (*P* < 0.05) (Fig. 8c,c’,q). The expression and intensity of MUC2-like-ir in the ESO did not differ significantly between the early and late stages (*P* > 0.05) (Fig. 8r). The intensity levels of MUC2-like-ir in the CD and PY regions were highest during the late stages of ovarian development, compared with early stages (*P* < 0.05) (Fig. 8e,e’,f,f’,r).

In the MGP section, the occurrence and intensity of MUC19-like-ir in the MGP was notably highest of the Ep and Ct layers in the late stages, compared with the early stages (Fig. 8g,g’,s). Nevertheless, the intensity of MUC19-like-ir in the MGD did not differ significantly between the early and late stages (*P* > 0.05) (Fig. 8h,h’,s). The MGP and MGD showed the highest expression and intensity of MUC2-like-ir in the apical part of the MGCs, MGCs and Ct in stage IV (*P* < 0.05), while the lowest intensity of MUC2-like-ir was seen in early stages (Fig. 8k,k’,l, l’,t). The expression and intensity level of MUC19-like-ir in the HGVi and HGG were notably greater during the early stages than the late stages of ovarian development (*P* < 0.05) (Fig. 8i,i’,j, j’,s). Furthermore, the HGVi in the late stages exhibited the highest intensity of MUC2-like-ir, while the early stages showed the lowest intensity (*P* < 0.05) (Fig. 8m,m’,t). In the HGG, there was a significantly higher intensity of MUC2-like-ir at the late stages, compared with the early stages (*P* < 0.05) (Fig. 8n,n’,t).

## Discussion

We are the first to provide a comprehensive description of the detailed morphological, histological, histochemical structures, and their alterations in the FG, MG, and HG during the ovarian cycle of female *M. rosenbergii*. The FG and HG are lined by the Ep with a Cu layer, whereas the MG does not have this layer. The FG and MG display the thickest striated Ms layer, while the HG has the thinnest Ms layer. The reactivities of PAS/AB in these digestive organs changed throughout the ovarian cycle, and this was supported by the increased expression of mucin levels as ovarian maturation progressed. Our findings provide novel and important insights into a significant correlation between anatomical and histochemical alterations of the digestive organs, feeding, and the ovarian cycle in the female prawn, *M. rosenbergii.*

### Morphological, histological, and histochemical structures of FG and their changes during the ovarian cycle in female prawns, *M. rosenbergii*

The female *M. rosenbergii* ESO possesses a short, stretchable tube, and longitudinal folds that link the Mo and CD parts. The Ep cell types of the ESO in *M. rosenbergii* consist of the pseudostratified columnar epithelium with a chitinous Cu layer attached to a Bm. In the crab, *Maja brachydactyla*, the ESO wall contains several longitudinal folds, which helps enlarging the surface area of the ESO to facilitate the movement and digestion of food before passing into the CD chamber (Castejón et al. 2018). In the shrimp, *Metapenaeus bennettae*, and *Palaemonetes argentinus*, the foldings in the wall of the ESO may facilitate its expansion and peristalsis during food ingestion (Dall 1967; Sousa and Petriella 2006; Brösing 2010). In the prawn, *Palaemonetes argentinus*, and the crab, *Menippe rumphi*, the ESO is lined with a simple columnar epithelium covered by the Cu (Babu et al. 1982; Sousa and Petriella 2006). In contrast, the ESO of crab, *Pseudocarcinus gigas*, is made up of cuboidal epithelial cells covered by the Cu (Heeren and Mitchell 1997). This system is involved in grinding food and provides points of Ms attachment, to facilitate mechanical digestion (Brösing 2010). Thus, the characteristics of the ESO of *M. rosenbergii* is similar to that described in *M. brachydactyla* (Castejón et al. 2018), and *M. bennettae* (Brösing 2010). We also observed a broad and dense circular muscle (CM) that is connected to longitudinal striated muscles (LM) in close proximity to the Ep of the ESO in female *M. rosenbergii*. The histological pattern of Ms in the ESO is similar to those observed in the crab *M. brachydactyla* (Castejón et al. 2018), the lobster *Nephrops norvegicus* (Yonge 1924), and the European lobster *Homarus gammarus* (Barker and Gibson 1977). In *M. brachydactyla*, the presence of the Ms layers could be related to the peristaltic action that is involved in the process of food ingestion (Castejón et al. 2018). Thus, the enlargement of the ESO and the rhythmic Ms contraction during ingestion and swallowing in female *M. rosenbergii* may be modulated by these Ms, which contribute to the peristaltic motion involved in food ingestion and mechanical breakdown.

In female *M. rosenbergii*, the Cu comprises the Ec, PC, and Mb layers. The Pc is the thickest and most densely calcified layer, whereas the Mb is the innermost layer that is in contact with Ep cells. These features are similar to those reported in the mud crab *Scylla serrata* (Barker and Gibson 1978), and crab *Burytelphusa cuniculuris* (Diwan 1972). In the crayfish, *Procambarus clarkii,* the adluminal Ep is coated with the Cu, consisting of the Ep, Ex, and Ed layers. The Ex is a rigid and located at the top along the apicolateral surfaces of the Ep, and the Ex becomes thinner on the sides of the folds (To et al. 2004). In adult *M. brachydactyla*, the Cu contains the three layers, and the Ep is the outmost Cu layer, while both the Ex and Ed layers are distinct from the Ep layer because of their lamellar structure and their homogenous staining affinity (Castejón et al. 2018). In crustaceans, the Cu is composed of the polysaccharide chitin, which is made up of monomers of *N*-acetyl-glucosamine. In addition to chitin, the organic matrix of Cu also consists of glycoproteins, lipids, and colors (Dillaman et al. 2013; Mrak et al. 2017). The Cu exhibits permeability to both water and salts, allowing the transport of water and ions (Mykles 1979; Johnson 1980; To et al. 2004). Hence, the components of Cu layer are conserved among decapod crustaceans, including those in female *M. rosenbergii.*

The ESOG of female *M. rosenbergii* contains a cluster of GCs that are organized into an acinar-like structure, and these GCs surround a CH. The ESOG secretes mucus that may help to protect the Ep cells from degradation by proteolytic enzymes (Vogt 2021). The *H. americanus*, possesses tegmental glands that secrete a waxy material, and release acid mucopolysaccharides (MPS) to lubricate its mouthparts (Felgenhauer 1992; Smolowitz 2021). In *H. gammarus*, the ESOG consists of MPS, acid phosphate, and ATPase (Barker and Gibson 1977). The gland of *M. brachydactyla* resembles salivary glands, which produce acidic mucus that aids in lubricating the intestinal lumen during digestion (Hunt et al. 1992; Castejón et al. 2018). Therefore, the ESOG of female *M. rosenbergii* may also produce and release mucus to lubricate its inner surface and protect the Ep from digestive enzymes. This was supported by the presence of strong AB/PAS reactivities and a high expression of both MUCs-like-ir in the GCs, reflecting the high secretion of mucus, as well as the existence of thick bundles of collagen fibers to reinforce the gland during the mucin synthesis and secretion.

The CD in female *M. rosenbergii* is lined with a pseudostratified columnar epithelium with a thick Cu coating. The CMs and LMs of the CD are well-developed and surrounded by the Ct. The mucosal layer of the CD is generally similar to that of the ESO. In some decapod crustaceans, the gastric mill (GM) rhythm controls the movement of the two lateral teeth and the single medial tooth (Marder and Busher 2007). In the shrimp, *Crangon septemspinosa,* the FG became more intricate, developing a stomach with lamellae as the larvae grew, and this stomach forms the GM, which is located between the pyloric and cardiac zones (Reynault 1972; Ceccaldi 1989). In the shrimp, *Litopenaeus vannamei* and *L. setiferus*, the GM is not present in any of the larval stages (P1-P10) and post-larval stages (Lovett and Felder 1989; Muhammad et al. 2012). In many Caridians, the GM is reduced in size or absent in older stages (Ceccaldi 1989). The Brazilian native prawn, *M. carcinus*, also lacks the GM and instead relies on the presence of sand in its diet to aid in food pulverization, however, the CD functions as a GM and is responsible for breaking down large pieces of food (Lima et al. 2014; 2016). Collectively, the absence of the GM in female *M. rosenbergii*, suggests species-specific pattern among decapod crustaceans.

We observed small spines, pores, and duct-like structure in the Cu of FG in female *M. rosenebrgii*. In grasshoppers and crickets (Orthoptera: Saltatoria), the Cu surface has numerous small hair-like structures, namely microspines (MP) (Elzinga and Hopkins 1995; Castejón et al. 2018). These MPS are present in ectoderm derivatives, such as the FG and HG (Elzinga and Hopkins 1995). In *Panulirus ornatus*, the short spines were present in the ESO lumen of the larval stages (Johnston et al. 2008). In *M. brachydactyla*, the surface of the Cu contains MPS and pores. The MPS extend from the Ep in the shape of filamentous structures, suggesting that they may assist in capturing and consuming food (Castejón et al. 2018). The pores in the Cu of FG of *M. brachydactyla* are somewhat elevated, compared with the surrounding the Cu (Castejón et al. 2018). There are pores in areas of the Cu located above the rosette glands, indicating releasing of the gland secretions (Elzinga and Hopkins 1995; Castejón et al. 2018). In *H. gammarus*, both small and large pores in the ESO were observed (Robertson and Laverack 1979). In female *M. rosenbergii*, the features observed resemble those documented in *M. brachydactyla* (Castejón et al. 2018), and *Homarus* sp. (Johnston et al. 2008). However, ultrastructural examinations are required to investigate the fine structures of the MPS and pores in female *M. rosenbergii*.

The ESO, CD, and PY portions exhibited strong PAS reactivity in the Ec and PC layers of Cu, whereas weak AB reactivity was observed. Nevertheless, we observed no PAS reactivity in the Mb layer, despite its strong AB reactivity. Considering the staining properties by hematoxylin and eosin (H&E) and PAS/AB in relation to the surrounding structures, it is likely that the substances in the MB layer may be secreted by the Ep cells. On the other hand, the Ec and proCu exhibited a strong PAS reaction, indicating the presence of high acid MPS rich in glycoprotein (Štrus et al. 2019; Vogt 2021). The PY of the female *M. rosenbergii* has a simple columnar epithelium, and contains a thinner Cu layer, compared with the CD. The PY has several longitudinal ridges and villi (Vi), and contains the inner oblique-oriented LM and the outer CMs.

The *P. clarkii* contains a simple columnar Ep and is encircled by the Cu layer. The CM and LM control the base of each ridge in the PY, suggesting that these Ms aid in the flexion of a chamber where ingested food is pulverized (To et al. 2004). The distinctive twisting (torsional) structure of peristaltic waves observed in *P. clarkii* may be attributed to the oblique orientation of the LMs (Brenner 1999). Thus, the characteristics of Ep cells and two Ms layers of the PY in female *M. rosenbergii* are similar to that observed in *P. clarkii* (To et al. 2004), and the presence of Vi in the PY may enhance the absorption of nutrients and facilitate the rhythmic contraction waves.

We found alterations in the histological and histochemical structures of the ESO during the ovarian cycle in female *M. rosenbergii*. In the late stages, the height of the Ep cells and the number of cells in the ESO were greater than the early stages. The higher Ep cells and their increased number in the late stages may indicate higher Ep cell activity in response to increased food intake, increased digestive enzyme activity, to satisfy the high requirement for nutrients to promote vitellogenesis and ovarian maturation. A strong AB reaction and a significant expression of MUC also support this speculation. However, the width and height of the villi, as well as the depth of the crypt appeared to be larger in the early stages. It is possible that the activity of Vi of the PY seems to be prominent in the early stages, which may be crucial for increasing the absorption of nutrients during periods of high food intake to promote growth (López-Greco and Rodríguez 1999; Bo et al. 2020). The expression of *MUC* mRNA level and the expression of MUC2-like-ir and MUC19-like-ir in the Cu and GCs in the FG parts were considerably higher in the late stages than the early stages in female *M. rosenbergii*, which were well correlated with the thickness of the MUC layer. MUCs are the major components of the mucus and are essential for protecting the digestive organs from digestive enzymes and enteric pathogens (Ogata et al. 2017; Duan et al. 2018; Murmu et al. 2023). In aquatic animals, including fish and crustaceans, previous studies have reported that MUC19 and MUC2 in the intestine have roles in nutritional digestion and absorption, mucus production, maintaining the intestinal barrier, and stimulating the innate immune system (Wang et al. 2012; Duan et al. 2018). In the crab, *Hemigrapsus nudus*, both the Cu and the digestive gland contain the acid MPS comprising glucose, galactose, and fucose residues. The concentration of MPS is higher in the Cu, where it is the sole soluble MPS, compared to the digestive gland (Meenakshi and Scheer 1959). Our findings in female *M. rosenbergii*, showed that the Cu contains high amount of MPS as reflected by PAS/AB reactivities, which was associated with high expression of MUCs-like-ir in this area, to help lubricate the lumen, sealing food, and protecting against pathogenic infections.

### Morphological, histological, and histochemical structures of the MG and their changes during the ovarian cycle of female prawns, *M. rosenbergii*

The MG in female *M. rosenbergii* is a straight cylindrical structure extending from the abdomen to the anus. The MGCs constitute the Ep layer of the MG, and are connected to the conspicuous BM. Notably, the Mv and subapical vacuoles were abundantly present on the apical surface of the MGCs. However, the MG epithelium does not have Cu. The MG in decapod crustaceans is derived from the mesenteron, whose derivative lacks Cu on the adluminal Ep (Mykles 1979; Trinadha Babu et al. 1989; To et al. 2004). The Ep of the MG consists of absorptive cells and may also include ionoregulatory cells (Mykles 1979; Štrus et al. 2019), and the MG cells (MGCs) secrete digestive enzymes and a protective chitinous material, called the peritrophic membrane (PM). The PM covers villi, and is made up of chitin, along with glycoproteins and proteoglycans that are believed to be deposited by the MGCs (Dall 1967; Georgi 1969; Wang et al. 2012). The MGCs of decapod crustaceans possess abundant RER (rough endoplasmic reticulum), Golgi bodies, and secretory vesicles, suggesting their potential role in the synthesis of the PM (Vogt 2021). This PM protects the cells from abrasion and damage caused by ingested food (Hegedus et al. 2009; Lehane and Billingsley 2012), and facilitate movement of a faecal string toward the anus with each successive cycle of peristalsis (Erri Babu et al. 1982; To et al. 2004). The subapical vacuoles of the MGCs have a role in absorbing nutrients from the tubular lumen (Vogt 2021). In *Macrobrachium carcinus*, MGCs contain the subapical vacuoles on the apical surface and they have well-developed brush borders, suggesting that the MGCs may promote absorption (Ruiz et al. 2020). In *H. americanus* and *C. magister,* the MGCs have a large number of mitochondria and Mv on the cell apices to enhance absorption (Ahearn and Maginniss 1977; Felder and Felgenhauer 1993). These MGCs possess acid MPS on the brush border, acting as surface binding molecules on the cell membrane and nutrient absorption (Rangneker and Momin 1974). These characteristics were also present in other shrimp species, including *Palaemon elegans* and the crayfish, *Astacus astacus* (Vogt 2021). Thus, the organization of the MG in female *M. rosenbergii* is similar to those found in other decapod crustaceans. MGCs may play a role in the absorption and transportation of nutrients and metabolites across membranes. This was supported by the strong MUC expression and significant reactivities of PAS/AB in the MGCs and Mv.

In H & E stained and semithin sections of female *M. rosenbergii* a distinct strongly PAS-positive Bm layer at the boundary between the Ep and subEp-Ct layers was observed. This feature is similar with other decapod species, including *H. americanus*, *H. gammarus*, *Cancer magister*, *Portunus sanguinolentus,* and *M. carcinus* (Trinadha Babu et al. 1989; Factor 1995; Ruiz et al. 2020). The Bm layer in the MG is a mesenteron-derived intestine, which helps to prevent excessive gut distension during food ingestion and flexibility of the viscus (Factor 1981; To et al. 2004). The striated Ms layer covering the MGP region in female *M. rosenbergii*, contains only well-developed inner CMs located beneath the Bm. Notably, in the MGP, there are bundles of collagen fibers with dense strips of elastic fibers in place of the outer LM layer. By contrast, in the MGD part, the muscle layer comprises mainly the obliquely oriented LM layer. These arrangements were clearly identified in the semithin and VGG stained sections. In adult *M. brachydactyla*, the MG caeca are surrounded by a thin circular Ms fibers (Castejón et al. 2022). In the prawn *M. carcinus,* a circular Ms layer is coved with a dense layer of loose Ct (Ruiz et al. 2020). The Ms of the MG are believed to provide support for the Ep cells, while the elasticity of the Ep layer is maintained by elastic fibers to facilitate the passage of food to the HG, as previously reported (Icely and Nott 1992; Conklin 1995; Factor 1995). The arrangement of Ms and Ct in the MG of female *M. rosenebrgii* seems to be different from those of *M. carcinus* and *M. brachydactyla*, suggesting that it may be a unique pattern specific to this species.

We have observed changes in histological and histochemical characteristics the MG of female *M. rosenbergii* during the ovarian cycle. The height of Ep cells of the MGP appeared to be increased throughout the mid-ovarian stages, and the numbers of cells were greater during the late stages. In worker honey bees, *Apis mellifera*, vitellogenin (Vg) has been identified in the MGCs (Harwood et al. 2019; Harwood and Adam 2021). In ticks, *Haemaphysalis longicornis*, MGCs Vg was detected and found to stimulate the ovaries, and facilitate the transportation of necessary nutrients from the blood meal (Harwood and Adam 2021; Thompson et al. 2007; Boldbaatar et al. 2010). The MG may maintain a consistently high level of Vg to facilitate its transport to developing eggs in the majority of oviparous animals (Pan et al. 1969; Raikhel and Lea 1983). Therefore, there is a correlation between the increased height of Ep cells and a greater number of AB positive cells in the MGCs at the late stages, and an enhanced capacity for food absorption to gain high nutrients and energy to promote ovarian maturation. This was found to be associated with the increased levels of Vg during the process of ovarian maturation in female *M. rosenbergii* (Jayasankar et al. 2020; Tinikul et al. 2023).

Strong expression of MUC2-like-ir and MUC19-like-ir was observed in the apical portions of the MGCs in the MGP of female *M. rosenbergii* during the late stages, compared with the early stages. The outermost layer of MGCs is covered by mucus layer, with mucin being the main component of the mucus, to lubricate the lumen, absorbs nutrients, and acts as a protective barrier against hazardous substances (Hartmann et al. 2013; Saborowski 2015; Murmu et al. 2023). In decapod crustaceans, the presence of mucin-like PM has been reported in *H. gammarus*, and *S. serrata* (Barker and Gibson 1978), and black tiger shrimp, *Penaeus monodon* (Soonthornchai et al. 2010). Collectively, these findings proposed that the MG may be the main secretory site of mucin secretion for the formation of the PM, to facilitate the passage of food, and protect intestinal Ep cells from pathogens (Perez-Villar and Hill 1999; Soonthornchai et al. 2010; Vogt 2021).

### Morphological, histological, and histochemical characteristics of the HG and their changes during the ovarian cycle of female prawns, *M. rosenbergii*

The HG of female *M. rosenbergii* contains the HGVi which are distinguished by their small size with only a thin Cu layer, and the Ep is lined by simple columnar cells formed by the HGCs. The HG is thought to play a role in the storage, lubrication, and movement of feces (Erri Babu et al. 1982; Devi et al. 1989). The HG is derived from the proctodeum and is lined with a chitinous Cu, which is similar to the lining of the stomodeum (Mykles 1979; Factor 1995; To et al. 2004). The Cu is known to contain high amount of MPS, therefore, it is possible that the tanning and formation of the Cu layer may also involve high levels of MPS secrete from in the tegmental glands or HGG (Dall 1967; To et al. 2004). The *H. gammarus* and *S. serrata* the HG is lined by a simple columnar epithelium, which forms longitudinal ridges and villi (Barker and Gibson 1977) In the lobster, *Panulirus argus*, there are numerous mitochondria in the apical cytoplasm of the HGCs, indicating their role in the absorption of ions from the gut lumen (Komuro and Yamamoto 1968; Malley 1977). In *H. americanus* and *H. gammarus*, the HGCs have a role in the transportation of ions and water. The absorption of fluid likely assists in the solidification and compression of the fecal strand (Mykles 1979). Therefore, the HG of female *M. rosenbergii* is similar to that of other decapod crustaceans, which primarily process and store fecal materials before expelling them to the exterior.

The musculature of the HG in female *M. rosenbergii* is composed of the outer CM and the inner slender bundles of LM fibers, extending towards the apices of the HGVi. These Ms extend into the longitudinal folds of the gut and are seen in the outer CM layers (Barker and Gibson 1977; Factor 1995). In *P. clarkii*, the LM bundles are essential for the twisting action, which enhances the strength of the villi in the HG and aids in mucus secretion (Brenner 1999; To et al. 2004; Castejón et al. 2021). Hence, the pattern of Ms in the HG of female *M. rosenbergii* is similar to that of other decapod crustaceans. The HGGs of female *M. rosenbergii* has a rosette-like arrangement containing serous and mucous cell types. A prominent AB-positive acid MPS was observed on the outermost surface of HGVi. We also noticed a mixture of elastic and collagen fibers surrounding GCs, which these likely to play a role in enabling the acini to stretch, and facilitate the release of secretions, and provide flexibility to the HG and anus (Mykles 1979; Trinadha Babu et al. 1989; Felder and Felgenhauer 1993). The HGGs serve as a temporary storage for intestinal contents prior to their entry into the HG (Rigdon and Mensik 1976; Muhammad et al. 2012). In *P. clarkii*, the mucous cells found in the intestinal glands share similarity with those found in the HGGs or tegumental glands of *Callinectes sapidus* and *H. americanus* (Johnson 1980; Factor 1995). In the crayfish *Astacus fluviatilis*, the HG is specialized for secretion and usually exhibits well-developed subepithelial tegmental glands (Smith 1978). The secretions of tegmental glands contain acid MPS, which are both sulphated and carboxylated in nature. However, some of the GCs also secrete neutral MPS (Erri Babu et al. 1985). In female *M. rosenbergii*, a strong positive AB reaction was noticed in the GCs at the early stages suggesting the presence of rich quantities of MPS, while mixed staining of AB and PAS was observed at the late stages, indicating the existence of both acid and neutral MPS. The acid mucous secretions in the GCs during the early stages may aid lubrication during digestion and enabling the prawns to obtain significant amounts of nutrients and energy. However, the occurrence of mixed types of mucous secretion during the late stages may be related to high activity in binding of fecal material and the facilitation its passage.

We found that MUCs-like-ir were present in the apical surface of the GCs lining HGCs during the mid- to late ovarian stages of female *M. rosenbergii*. Crustacean species exhibit rich quantities of MPS in their tegmental glands or HGG, which have been associated with the tanning process and the formation of the Cu layer (Dall 1967; To et al. 2004; McGaw and Curtis 2013). The HGG is involved in the generation of tyrosinase, which is associated with the formation of a novel Cu layer and the release of MUC. In insects, the PM is composed of chitin and proteins, mainly peritrophins. Peritrophins contain one or several chitin-binding domains (CBDs), which may have mucin-like domains (Tellam et al. 1999; Terra et al. 2019). The MUC protein derived from the tegmental glands or HGG, may assist in the attachment of oocytes and the strengthening of the egg cover in females (Talbot and Demers 1993). The high expression of MUCs in this HG area of female prawns, *M. rosenbergii*, may contribute to firm attachment of the oocyte and developed eggs during the spawning as reported in other crustaceans (Talbot and Demers 1993). Additionally, it may be involved in the production of the Cu and promotion of intestinal immune response (Vogt 2021). However, further studies are needed to investigate the potential functions of mucins and their mechanisms in female *M. rosenbergii.*

## Data Availability

The data that support the findings of this study are available from the corresponding author upon reasonable request.
